# Implementing an integrated inpatient–community rehabilitation pathway for adults hospitalised with acute exacerbations of COPD: a quality improvement study

**DOI:** 10.1136/bmjoq-2026-004550

**Published:** 2026-11-10

**Authors:** Peter Beshara, Clare Chung, Simon Miller, Emily Mackinlay

**Affiliations:** 1 Prince of Wales Hospital and Community Health Services, Randwick, New South Wales, Australia; 2 University of New South Wales, Sydney, New South Wales, Australia

**Keywords:** Patient Reported Outcome Measures, Rehabilitation, Transitions in care, Quality improvement, PDSA

## Abstract

**Introduction:**

Hospitalisation for acute exacerbations of chronic obstructive pulmonary disease (AECOPD) is associated with functional decline through acute illness, inactivity, deconditioning and loss of independence, while delayed rehabilitation and fragmented postdischarge care can disrupt recovery. We evaluated a physiotherapy-led integrated inpatient-to-community rehabilitation pathway designed to improve recovery timing and continuity.

**Methods:**

We conducted a prospective quality improvement initiative with retrospective quantitative evaluation of routinely collected process and patient-level outcome data. The Accelerated Respiratory Care (ARC) programme combined enhanced inpatient rehabilitation with an 8-week community pathway and was refined through three Plan–Do–Study–Act cycles. Consecutive eligible AECOPD admissions were screened. Functional and patient-reported outcomes were assessed at admission, discharge and 8 weeks. Admission to 8-week changes among participants with paired data were analysed using paired t-tests; process measures were examined using chronological run charts.

**Results:**

Of 310 AECOPD admissions, 81 enrolled and 47 provided complete admission to 8-week follow-up. Mean time to ARC initiation was 2.9 days. Functional performance improved (1-minute sit-to-stand +5.9 repetitions, 95% CI 4.2 to 7.6; Short Physical Performance Battery +2.3 points, 95% CI 1.6 to 3.0), exceeding published minimal clinically important differences. St George’s Respiratory Questionnaire Total improved by 6.5 points (95% CI −11.1 to −1.9); Symptoms and Impact improved significantly, while Activity did not. Multiple Patient-Reported Outcomes Measurement Information System-29 domains also improved significantly. No exercise-related adverse events occurred. Run charts showed non-random variation, including late increasing trends in community contacts and total programme exposure.

**Conclusion:**

ARC was feasible within routine care and associated with clinically meaningful improvements in functional and patient-reported outcomes among participants with complete follow-up. The pathway may provide a model for improving rehabilitative continuity after AECOPD, with the longer-term potential to reduce avoidable hospital use. Its effects on length of stay, readmissions and costs remain unestablished and require controlled evaluation. Sustained delivery is likely to require dedicated inpatient and community workforce capacity.

**WHAT IS ALREADY KNOWN ON THIS TOPIC**

**WHAT THIS STUDY ADDS**

- This quality improvement study demonstrates that a physiotherapy-led, integrated inpatient-to-community rehabilitation pathway can be implemented and iteratively refined within routine acute care, with clinically meaningful functional improvements among participants with complete 8-week follow-up and no exercise-related adverse events.

**HOW THIS STUDY MIGHT AFFECT RESEARCH, PRACTICE OR POLICY**

- The Accelerated Respiratory Care model may inform integrated inpatient-to-community rehabilitation to reduce avoidable hospital use. Controlled studies are needed to establish effects on length of stay, readmissions and cost-effectiveness, while sustainability likely requires adequately resourced inpatient and community respiratory services.

## Background

 Acute exacerbations of chronic obstructive pulmonary disease (AECOPD) are a common driver of hospital demand, associated with recurrent admissions, increased morbidity and substantial use of healthcare resources.^1–3^ In Australia, approximately $A1.8 billion in health-system expenditure was attributable to chronic obstructive pulmonary disease (COPD) in 2023–2024, with hospital services accounting for 77% of expenditure.^4^ COPD-related potentially preventable hospitalisations accounted for an estimated $A797 million in expenditure during the same period.^5^ Despite advances in pharmacological management, many patients experience prolonged length of stay, early readmission and persistent functional decline following hospital admission.^2 6 7^ Approximately 20% of patients hospitalised for an AECOPD are readmitted within 30 days,^8–11^ with a proportion of readmissions considered potentially avoidable.^12^

Hospitalisation represents a critical period of vulnerability for people with COPD. In addition to the acute respiratory insult, patients are exposed to immobility, disrupted sleep, cognitive stressors and loss of independence, contributing to hospital-associated disability (HAD).^13–15^ HAD is characterised by new or worsened impairments in physical function, mobility and activities of daily living, with effects that may be prolonged and recovery often incomplete.^16^ These effects are amplified in older adults and those with frailty and multimorbidity, characteristics common in AECOPD admissions.^16–18^

Pulmonary rehabilitation (PR) is an essential component of contemporary COPD care, with evidence showing benefits for exercise tolerance, symptom burden and health-related quality of life.^19^ Guidelines recommend referral to PR within 3–4 weeks following hospitalisation for AECOPD.^20 21^ Nevertheless, real-world uptake is often low or delayed, with some estimates suggesting fewer than 10% of eligible patients access PR after hospitalisation.^22–24^ Recent evidence indicates that rehabilitation initiated during or shortly after hospitalisation is feasible and safe,^25–27^ and transitional care interventions bridging the hospital–community interface have shown promise in improving continuity and reducing avoidable healthcare use.^28 29^ However, routine implementation of integrated rehabilitation pathways remains limited by workforce constraints, fragmented postdischarge services and inconsistent handover processes.^29–32^

The Accelerated Respiratory Care (ARC) programme was developed to address these gaps within routine care at a tertiary metropolitan hospital. ARC integrates enhanced inpatient rehabilitation with standardised assessment, formalised referral and structured transition to community-supported home-based exercise. The objectives were to examine the feasibility of embedding the pathway within routine care, describe iterative refinements through successive Plan–Do–Study–Act (PDSA) cycles and assess changes in implementation, functional and patient-reported outcomes.

## Methods

### Study design

We conducted a single-centre, quantitative quality improvement evaluation of a physiotherapy-led service intervention implemented prospectively between November 2023 and November 2024. ARC was introduced within routine clinical care and iteratively refined through three sequential PDSA cycles. Change ideas were introduced at service level in response to observed operational barriers rather than allocated at individual patient level. Clinical, process and patient-reported data were collected prospectively during programme delivery. After completion of the implementation period, these data were retrospectively analysed to describe implementation performance, changes across PDSA phases and patient outcomes. The evaluation was uncontrolled and used a single-arm, repeated-measures before-and-after design. It was designed primarily to assess feasibility, implementation and signals of improvement rather than to estimate a causal treatment effect. Reporting follows Standards for Quality Improvement Reporting Excellence (SQUIRE 2.0) guidance for quality improvement studies.^33^

### Setting and context

The study was conducted on an acute respiratory ward at the Prince of Wales Hospital (POWH), Sydney, Australia. Prior to ARC, inpatient rehabilitation was delivered through usual ward-based physiotherapy, with variable timing, intensity and continuity. Two system-level problems were identified: insufficient inpatient rehabilitation contributing to deconditioning and prolonged stays, and fragmented discharge transition producing poor postdischarge rehabilitative continuity.

ARC was embedded within routine service delivery and overseen through a working group linked to hospital governance. Inpatient delivery was supported by existing ward physiotherapy staffing and, from PDSA cycle 2 onwards, supervised physiotherapy students. The postdischarge phase was delivered by experienced community respiratory nursing and physiotherapy clinicians.

### Patient and public involvement

A brief preimplementation survey of 10 inpatients admitted with AECOPD was conducted prior to programme development, exploring experiences of hospitalisation, barriers to rehabilitation participation and preferences for inpatient exercise and postdischarge support. Findings directly informed key ARC design features: additional supervised exercise sessions, group-based delivery and structured postdischarge continuation. This process aimed to align programme design with patient priorities and improve acceptability and engagement across the inpatient-to-community care continuum.

### Intervention

#### Enhanced inpatient rehabilitation

Eligible patients commenced ARC within 72 hours of admission or when deemed medically stable by the respiratory team. In addition to routine ward-based physiotherapy, participants received daily supervised exercise sessions in a designated respiratory ward gym. Sessions were delivered in small groups by a senior respiratory physiotherapist (18 years’ experience) with support from supervised physiotherapy students from PDSA cycle 2 onwards. Exercise was individually tailored to functional capacity, symptom burden and clinical status. The inpatient programme (online supplemental file A) included upper and lower limb endurance, resistance and functional training consistent with PR principles. Intensity and progression were adjusted daily according to patient tolerance and clinical response.

#### Community respiratory follow-up and home-based exercise

At hospital discharge, all participants were referred to the Respiratory Coordinated Care Programme (RCCP), the local community respiratory follow-up service. Postdischarge contact was delivered by experienced respiratory nursing and physiotherapy clinicians via telephone or home visits, with contact frequency tailored to individual need and operational capacity. Participants received a structured home exercise programme for up to 8 weeks, comprising a walking programme and upper and lower limb strengthening exercises (online supplemental file B). A standardised ARC home exercise booklet, introduced in PDSA cycle 3, improved prescription consistency.

### Participants

Consecutive adult patients admitted with a primary diagnosis of AECOPD were screened for eligibility. Only the first eligible admission per patient was included in the evaluation. Inclusion required confirmed COPD (forced expiratory volume at 1 s/forced vital capacity <0.7 on prior spirometry) and medical stability for exercise. Patients were excluded for exercise contraindications, significant cognitive impairment limiting capacity to follow instructions or functional dependence precluding gym-based participation. Referral was made by respiratory medical, physiotherapy or senior nursing staff. Patients outside the POWH catchment were excluded when active follow-up could not be verified, as mailed questionnaires alone could not confirm programme completion.

### PDSA cycles

The improvement methodology was used to identify local barriers, test changes to service delivery, review process feedback and adapt the pathway. The intervention evolved across three sequential PDSA cycles, allowing refinement of processes, staffing models and postdischarge monitoring mechanisms in response to real-world operational barriers. Change ideas were implemented at service level; participants were not randomised or individually allocated to particular pathway components. A driver diagram (figure 1) linked the overall programme aim to key system drivers and the change ideas tested across cycles. The principal change ideas, data used to inform each cycle and resulting adaptations are summarised in online supplemental table 1.

**Figure 1:**
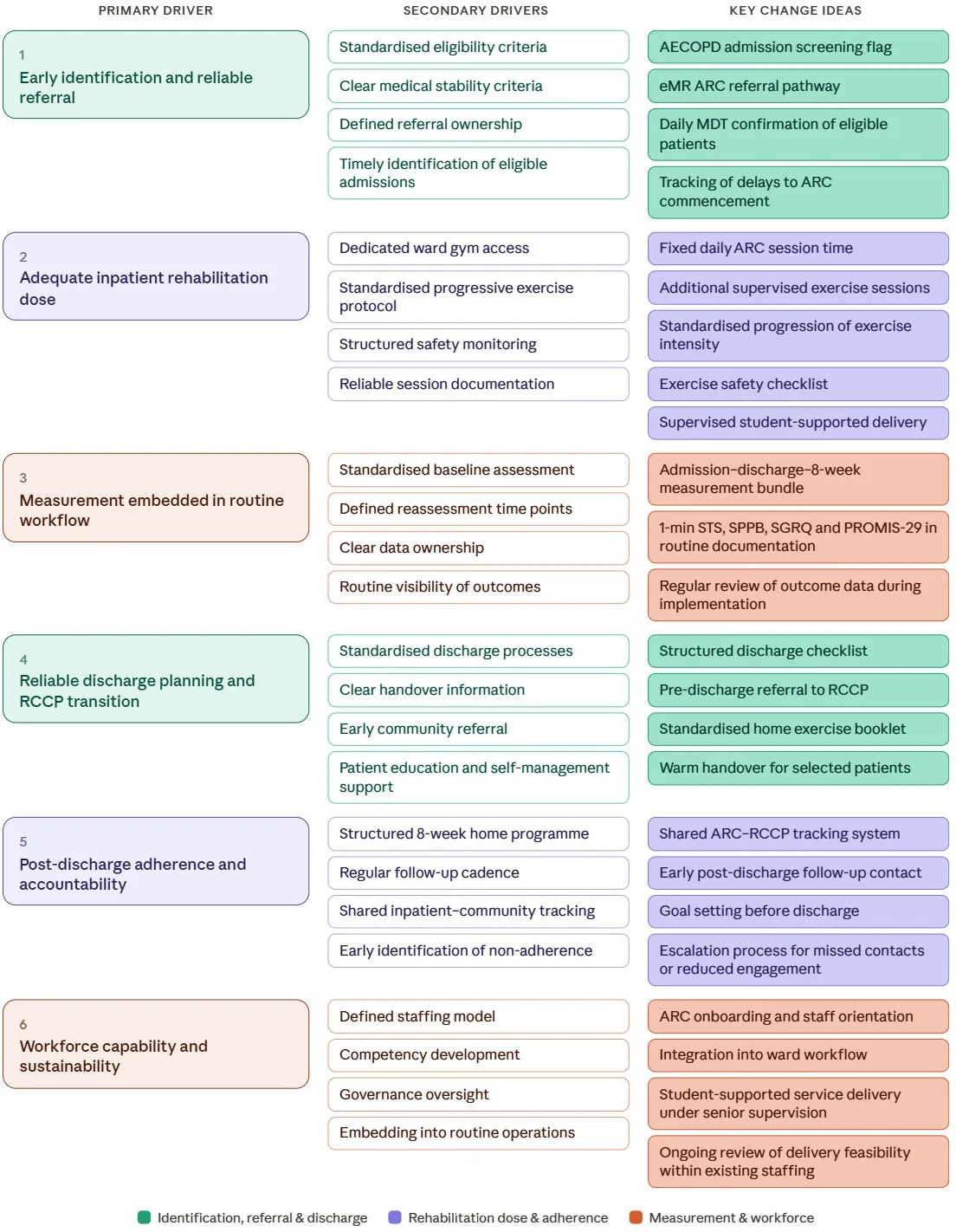
Driver diagram for the ARC programme. Driver diagram showing the overall programme aim, key system drivers and change ideas used to guide iterative implementation of the ARC inpatient–community rehabilitation pathway for patients admitted with acute exacerbation of COPD. AECOPD, acute exacerbations of chronic obstructive pulmonary disease; ARC, Accelerated Respiratory Care; COPD, chronic obstructive pulmonary disease; MDT, multidisciplinary team; eMR, electronic medical record; 1-min STS, 1-minute sit-to-stand test; PROMIS-29, Patient-Reported Outcomes Measurement Information System-29; RCCP, Respiratory Coordinated Care Programme; SGRQ, St George’s Respiratory Questionnaire; SPPB, Short Physical Performance Battery.

#### PDSA cycle 1 (November–December 2023)

Piloted feasibility, safety and acceptability of gym-based ARC sessions using existing physiotherapy staffing. Data collection focused on referral timing, session attendance and inpatient completion rates.

#### PDSA cycle 2 (January–August 2024)

Established a dedicated respiratory gym, standardised functional and patient-reported outcome measures (PROMs), integrated PROMs into the electronic medical record (eMR), formalised the electronic referral pathway to RCCP and incorporated supervised physiotherapy students into inpatient ARC delivery. Students participated in approximately 80% of inpatient ARC sessions under direct senior supervision, supporting session delivery, functional assessment and documentation.

#### PDSA cycle 3 (September–November 2024)

Targeted optimisation of postdischarge rehabilitation adherence and visibility of follow-up. A shared ARC–RCCP 8-week tracking spreadsheet was introduced to monitor attendance, exercise completion and follow-up contacts. A standardised ARC home exercise booklet was developed to improve consistency of postdischarge exercise prescription. Patient education prior to discharge was enhanced, with greater emphasis on goal-setting, expectations and the importance of ongoing rehabilitation participation. ARC patients were incorporated as a standing agenda item within existing weekly RCCP multidisciplinary safety huddles. After completion of ARC, RCCP clinicians reviewed the need for ongoing community respiratory involvement and continued follow-up where clinically appropriate.

### Measures

#### Process measures

Included time from admission to ARC commencement, enrolment rates, inpatient ARC sessions attended, community RCCP contacts received postdischarge, total programme dose (sum of inpatient ARC sessions and community RCCP contacts), outcome measurement completion and transition to RCCP.

### Outcome measures

Included the validated 1-minute sit-to-stand test^34^ (1-min STS) and Short Physical Performance Battery^35^ (SPPB) for functional performance; the St George’s Respiratory Questionnaire^36^ (SGRQ) for health-related quality of life; and Patient-Reported Outcomes Measurement Information System (PROMIS)-29^37^ for broader patient-reported health status. Outcomes were assessed at admission (following medical stabilisation, typically within 72 hours of ARC enrolment), discharge and 8 weeks postdischarge. Minimal clinically important differences (MCIDs) were defined as ≥3 repetitions for 1-min STS, ≥1 point for SPPB total score and ≥4 points for SGRQ Total score.

### Balancing measures

Included exercise-related adverse events and implementation burden within existing staffing structures, including pressure on inpatient and community teams, workflow disruption and sustainability challenges.

### Data collection and analysis

Data were collected prospectively during routine programme delivery from the eMR, ARC clinical logs and RCCP tracking processes. PROMs were captured through the NSW Health Outcomes and Patient Experience (HOPE) platform, an eMR-integrated system used locally for routine collection of patient-reported outcomes and experiences. Patients completed PROMs on paper or hospital-issued iPads, with responses entered or uploaded into HOPE as part of clinical workflow. The first eligible admission per patient was included. Following the implementation period, the dataset was retrospectively analysed for this evaluation. Online supplemental table 2 summarises the recorded variables, data sources, timing and intended use in the evaluation.

Because programme delivery evolved across PDSA cycles and community follow-up intensity varied with staffing availability, a uniform intervention dose could not be defined across all participants. The primary outcome analysis used a complete-case, repeated-measures approach and included participants with paired outcome data from admission to 8 weeks postdischarge. Baseline demographic and clinical characteristics were summarised using mean±SD or median (IQR) for continuous variables, according to distribution and number (%) for categorical variables. Programme-participation variables, including time to ARC commencement, inpatient ARC sessions, community RCCP contacts and total programme dose, were summarised descriptively overall and by PDSA cycle. Completers and non-completers were compared using Mann-Whitney U tests for continuous variables, Fisher’s exact tests for binary categorical variables and χ² tests for categorical variables with more than two levels.

Patient outcomes were analysed using paired observations for the relevant time points. No missing values were imputed; participants contributed to an outcome analysis only when data were available at both time points required for that comparison, and outcome-specific denominators are reported. Discharge measurements were summarised using available observations and were not carried forward or otherwise imputed. No assumption was made that missing outcome data were completely at random.

Admission to 8-week changes were assessed using paired t-tests, with mean differences, 95% CIs and two-sided p values reported. Cohen’s d effect sizes were additionally reported for the functional outcomes. Statistical significance was defined a priori as p<0.05. Analyses were exploratory, and p values were not adjusted for multiple comparisons. Clinically meaningful change was interpreted using published MCIDs. Analyses were conducted using SPSS V.31.

Process measures were examined using chronological run charts to distinguish descriptive variation from potential non-random change. Participants were ordered by hospital admission date, and the timing of PDSA changes was annotated. Because an adequate preintervention baseline series was not available and PDSA cycle 1 comprised only nine observations collected during active implementation, a single median calculated from the full 47-participant series was used as a fixed centreline for retrospective signal detection. This was a pragmatic approach in the absence of a sufficiently long preintervention baseline from which to establish and freeze a baseline median.

Run charts were interpreted using the rules described by Perla *et al*^38^: a shift was defined as six or more consecutive observations above or below the median; a trend as five or more consecutive observations increasing or decreasing; and an unusually low or high number of runs as a value outside the expected limits for the number of observations not falling on the median. An astronomical point was defined as an observation clearly and visibly distinct from the remainder of the series. Observations falling on the median were excluded when assessing shifts and the number of runs. For trend assessment, consecutive identical values were counted once and did not contribute to or interrupt a trend. Cycle-specific medians were retained as descriptive summaries but were not interpreted as evidence of non-random change.

## Results

### Participant flow and cohort characteristics

During the study period, 310 AECOPD admissions were screened; 81 patients enrolled in ARC and 47 provided complete outcome data from admission to 8 weeks postdischarge. The analytical cohort was accrued across all three PDSA cycles, with reasons for non-enrolment, attrition and incomplete follow-up shown in online supplemental figure 1.

### Comparison of completers and non-completers

Completers and non-completers did not differ significantly in age, sex, body mass index, COPD severity, most comorbidities, length of stay, 28-day readmission, prior hospitalisation or previous PR (online supplemental table 3). Two differences reached statistical significance: completers had a higher prevalence of coronary heart disease (26% vs 6%, p=0.035) and dyslipidaemia (32% vs 9%, p=0.016). Baseline functional performance did not differ significantly between groups: admission 1-min STS was 13.7±6.1 repetitions in completers versus 14.8±7.0 in non-completers (p=0.478), and admission SPPB total score was 7.1±2.5 versus 7.7±3.0 (p=0.362). Non-completers were marginally higher on both measures at baseline, indicating that the functional gains observed in completers are unlikely to reflect a systematic advantage in baseline physical capacity.

### Implementation and process outcomes

There were no significant differences between completers and non-completers in time to ARC commencement (mean 2.9±2.7 vs 2.9±2.4 days, p=0.763), nor in the proportion commencing within 72 hours of hospital admission (72% vs 65%; p=0.477). However, completers attended significantly more inpatient ARC sessions (median 3, IQR 2–3 vs median 1, IQR 1–2; p<0.001), more community RCCP contacts (median 6, IQR 3–10 vs median 2, IQR 0–6; p<0.001) and a greater total number of ARC sessions/contacts overall (median 9, IQR 5–12 vs median 4, IQR 2–6; p<0.001).

Run-chart analysis demonstrated non-random variation in time to ARC commencement (figure 2a). The overall median was 2 days. Among 35 useful observations, 11 runs were identified, below the expected lower limit of 12. An 11-useful-observation shift above the median occurred between February and March 2024, followed by an eight-useful-observation shift below the median between April and July 2024; observations falling directly on the median were excluded when assessing shifts. No five-point trend was identified. Cycle-specific medians were 1 day in cycle 1 (n=9), 2 days in cycle 2 (n=34) and 3.5 days in cycle 3 (n=4), with 78%, 74% and 50% commencing within the 72-hour target, respectively.

**Figure 2:**
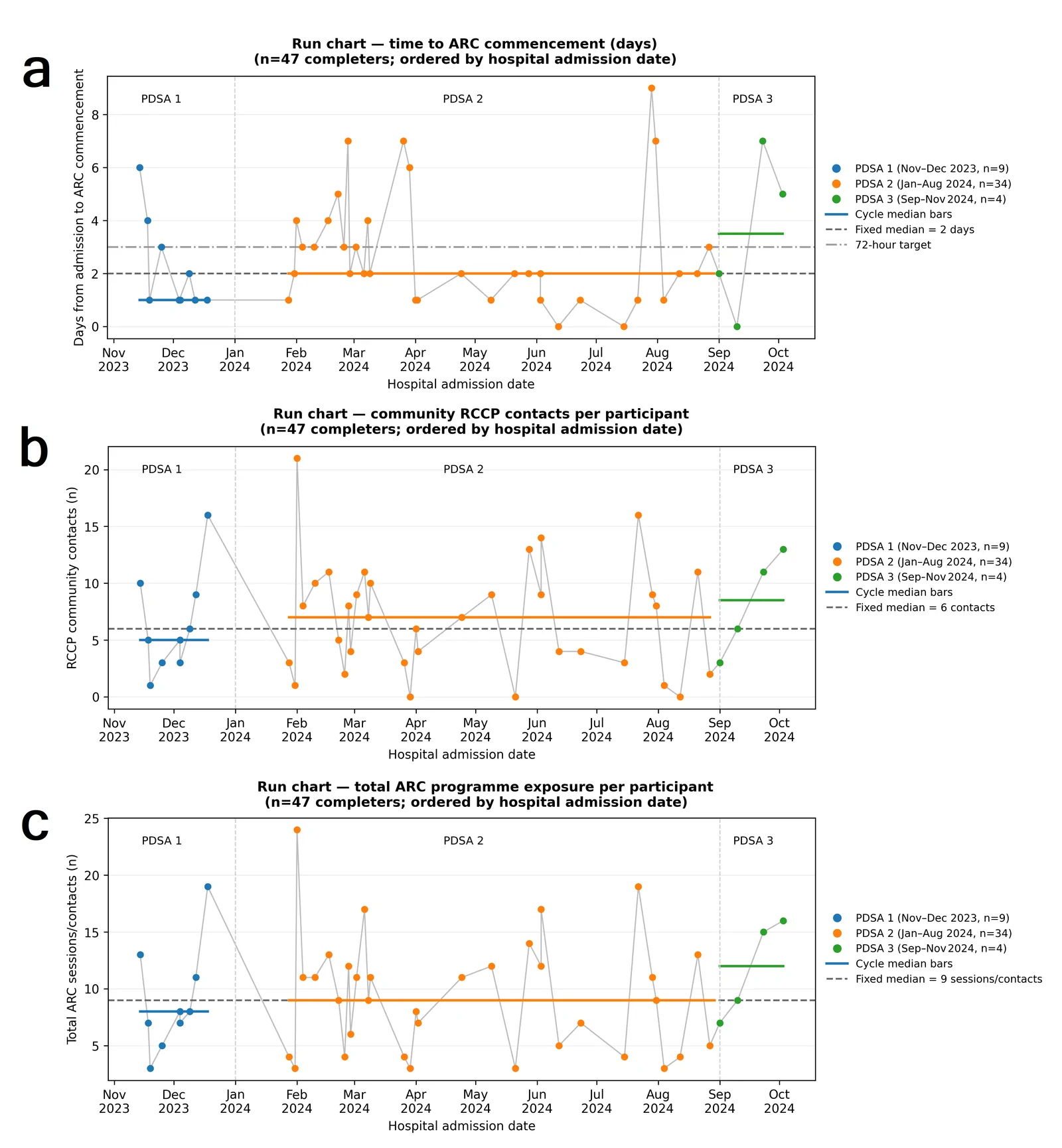
(a) Run chart—time to ARC commencement (days). Each data point represents an individual participant plotted chronologically by hospital admission date. (b) Run chart—community RCCP contacts per participant. Recorded RCCP contacts during the 8 week post-discharge period are plotted chronologically by hospital admission date. (c) Run chart—total ARC programme dose. Total ARC programme dose defined as the sum of inpatient ARC sessions and community RCCP contacts per participant. Data points ordered by admission date; horizontal bars represent cycle medians. ARC, Accelerated Respiratory Care; PDSA, Plan–Do–Study–Act; RCCP, Respiratory Coordinated Care Programme.

Community RCCP contacts had a median of six contacts per participant (figure 2b). 19 runs were observed among 44 useful observations, within expected limits and no six-point shift occurred. A five-point decreasing trend occurred between late July and mid-August, followed by a five-point increasing trend from late August to October 2024.

Total programme dose had a median of nine inpatient sessions/community contacts (figure 2c). 19 runs among 43 useful observations were within expected limits (16**–**28). An early six-point shift below the median occurred during the initial implementation period, while a five-point increasing trend occurred between late August and October 2024 (5, 7, 9, 15 and 16 sessions/contacts). No astronomical point was identified. Cycle-specific medians were 8, 9 and 12 sessions/contacts in cycles 1–3, respectively.

### Functional outcomes

Functional performance improved across the pathway (table 1, online supplemental figure 2). Mean 1-min STS increased from 13.7±6.1 repetitions at admission to 17.2±7.4 at discharge and 19.6±7.4 at 8 weeks. The change from admission to 8 weeks was +5.9 repetitions (95% CI 4.2 to 7.6, Cohen’s d=0.87, p<0.001), exceeding the MCID of ≥3 repetitions. 26 participants (55.3%) achieved a clinically meaningful improvement in 1-min STS by hospital discharge. Mean SPPB total score improved from 7.1±2.5 at admission to 8.4±2.4 at discharge and 9.4±2.1 at 8 weeks (change: +2.3, 95% CI 1.6 to 3.0, Cohen’s d=0.95, p<0.001), exceeding the MCID of ≥1 point.

**Table 1:** Functional performance at admission, discharge and 8 weeks postdischarge among ARC completers (n=47)

Outcome	Admission Mean±SD	Discharge Mean±SD	8 weeks Mean±SD	Change (Adm→8 w)	95% CI	Cohen’s d	P value
1-min STS (reps)	13.7±6.1	17.2±7.4	19.6±7.4	+5.9	4.2 to 7.6	0.87	**<0.001**
SPPB total (0–12)*	7.1±2.5	8.4±2.4	9.4±2.1	+2.3	1.6 to 3.0	0.95	**<0.001**

* Higher scores indicate better function. MCID ≥3 repetitions for 1-min STS; ≥1 point for SPPB total score. All p values from paired t-tests comparing admission with 8-week follow-up.

ARC, Accelerated Respiratory Care; MCID, minimal clinically important difference; 1-min STS, 1-minute sit-to-stand; SPPB, Short Physical Performance Battery.

### Health-related quality of life and patient-reported outcomes

Several health-related quality-of-life and patient-reported outcomes improved by 8 weeks postdischarge (online supplemental table 4). SGRQ Total score improved by 6.5 points (95% CI −11.1 to −1.9, p=0.006), exceeding the 4-point MCID. Symptoms and Impact domain scores improved significantly (p=0.013 and p=0.016, respectively). The Activity domain showed a numerical improvement of 4.7 points that did not reach statistical significance (p=0.118). Across PROMIS-29 domains, significant improvements were observed in Physical Function (+2.2, p=0.004) and Social Roles (+3.6, p=0.006), with reductions in Anxiety (−3.4, p=0.011), Depression (−3.1, p=0.018), Fatigue (−5.3, p=0.003), Sleep Disturbance (−2.9, p=0.041), Pain Interference (−3.4, p=0.015) and Pain Intensity (−0.8, p=0.032).

### Balancing measures

No exercise-related adverse events were identified during any phase of ARC delivery, including early mobilisation during acute admission, group-based gym sessions and student-supported inpatient delivery. Implementation burden was manageable during the inpatient phase but became more apparent in the community phase. Later PDSA cycles highlighted that ongoing high-quality postdischarge delivery relied heavily on senior RCCP physiotherapy input and was difficult to maintain alongside broader community service demands without additional resourcing.

### PDSA learning across cycles

Implementation learning broadly aligned with the priorities identified in the driver diagram, with referral reliability, inpatient delivery consistency, discharge transition and postdischarge adherence emerging as the principal determinants of pathway performance.

#### Cycle 1

Confirmed that medically stable patients could participate safely in group-based exercise during acute admission. Referral timing was variable, documentation inconsistent and postdischarge uptake had limited visibility. Patient engagement was high. In response, referral processes were clarified in collaboration with respiratory medical teams, and ARC was formally endorsed as a ward-level model of care rather than an optional adjunct to routine physiotherapy.

#### Cycle 2

Improved reliability of inpatient delivery through a dedicated gym space, fixed session timing, standardised outcome measurement and supervised student-supported delivery. Students assisted with group-based exercise sessions, progression and monitoring of individual exercise programmes, administration of functional outcome measures and documentation aligned with routine clinical workflows, under direct supervision of senior physiotherapists. Delivery volume and documentation consistency improved, though the student model required deliberate preparation and stable senior oversight.

#### Cycle 3

Strengthened postdischarge monitoring through a shared ARC–RCCP tracking spreadsheet, a standardised home exercise booklet, enhanced predischarge goal-setting and incorporation of ARC patients into RCCP multidisciplinary safety huddles. Earlier identification of non-adherence, postdischarge compliance barriers and clinical deterioration was observed. However, maintaining the required intensity of community physiotherapy follow-up alongside broader RCCP service demands proved challenging, supporting the recommendation that additional community physiotherapy or allied health assistant resourcing should be considered if the model is to be sustained.

## Discussion

This study evaluated a physiotherapy-led integrated inpatient-to-community rehabilitation pathway for patients hospitalised with AECOPD. Among participants who completed the full pathway, ARC was associated with clinically meaningful improvements in functional performance and patient-reported outcomes, with no exercise-related adverse events identified. Chronological process analysis demonstrated non-random variation during implementation rather than uniform progressive improvement: time to ARC commencement showed sequential shifts toward longer and then shorter commencement intervals, while community contacts and total recorded programme exposure demonstrated late five-point increasing trends. For us, the key learning was that rehabilitation after AECOPD could not be treated as a single inpatient intervention or a separate postdischarge referral. It needed to function as one connected pathway across admission, discharge and recovery at home.

PR is strongly supported in COPD management and timely postexacerbation referral is recommended,^20 21^ yet real-world data continue to show very low uptake after hospitalisation.^22–24^ In this context, ARC did more than provide additional exercise sessions; it reconfigured when, where and how rehabilitation occurred, embedding it earlier during admission and linking it explicitly to structured postdischarge continuation. This is clinically important because the early postexacerbation period is characterised by deconditioning, reduced confidence, impaired function and heightened risk of further deterioration.^13–18^ By aligning rehabilitation delivery with this period of vulnerability, ARC attempted to reduce the usual delay between recovery from the acute medical event and access to restorative care.

The large improvements and effect sizes for 1-min STS and SPPB at 8 weeks are consistent with evidence that early exercise-based rehabilitation during or soon after hospitalisation for AECOPD can produce clinically meaningful improvements in physical function.^25–27^ These findings must nonetheless be interpreted cautiously. Some improvement would be expected with resolution of the exacerbation itself, and the absence of a control group prevents firm causal attribution. The more plausible interpretation is that ARC may have supported, structured or consolidated recovery that would otherwise have been less consistent in routine care. This distinction is important in quality improvement work, where interventions modify conditions rather than act as isolated treatment effects.

The patient-reported findings also deserve nuanced interpretation. Improvements in SGRQ Symptoms, Impact and Total scores, together with gains across PROMIS-29 domains, suggest that ARC may have influenced broader dimensions of recovery beyond lower-limb performance. Recovery after AECOPD is multidimensional, encompassing breathlessness, fatigue, sleep disturbance, anxiety, depressive symptoms, social participation and self-efficacy.^13 14 18^ Exercise training may have contributed directly, but education, repeated therapist contact, structured follow-up and goal-setting after discharge may also have been important mechanisms. The absence of a statistically significant change in the SGRQ Activity domain is equally informative. Activity limitation after AECOPD is shaped by chronic dyspnoea, frailty, multimorbidity and environmental constraints that may not fully respond over an 8-week period, particularly in an older cohort with substantial baseline impairment.^15–18^ This pattern suggests that some domains are more responsive than others and that longer follow-up or more intensive community support may be needed to influence habitual activity.

From an implementation perspective, the study reinforces that outcomes in AECOPD are strongly shaped by system design rather than single therapeutic components. Early in the project, feasibility of inpatient exercise alone did not translate into a functioning pathway; performance was determined by referral reliability, documentation consistency, cross-setting communication and visibility of postdischarge engagement. This aligns with literature showing that integrated COPD care is often constrained less by lack of efficacy than by organisational fragmentation, workforce pressures and weak cross-setting coordination.^30–32^

The run-chart findings reinforce the need for caution when interpreting sequential PDSA data since descriptive differences between cycle medians are not themselves evidence of sustained improvement. The shifts in time to ARC commencement indicate that referral timeliness changed non-randomly during implementation but not in a uniformly favourable direction. The late increasing trends in community contacts and total recorded programme exposure are compatible with greater follow-up late in implementation, but they spanned the transition between cycles 2 and 3 and did not constitute qualifying shifts. The small final cycle and absence of a concurrent comparator therefore prevent attribution of these signals to any single change idea. ARC is best understood as an evolving service redesign intervention whose implementation effects require continued measurement over time.

The inclusion of supervised physiotherapy students into ARC inpatient delivery was a notable operational feature. Under senior supervision, students supported approximately 80% of inpatient ARC sessions, contributing to functional assessment and documentation without identified safety concerns. This is consistent with emerging evidence that supervised student-delivered rehabilitation can be feasible and safe,^39 40^ and physiotherapy student-led services are not perceived to compromise care quality or patient safety.^41 42^ However, these models require appropriate governance, supervision, infrastructure and resourcing, and their feasibility appears to be context-dependent rather than universally transferable.^43 44^ Our student-supported delivery served as a pragmatic adjunct to stable senior clinical leadership for the inpatient phase rather than a substitute for experienced staffing. The community phase, meanwhile, remained dependent on established RCCP respiratory clinicians to deliver safe and consistent postdischarge follow-up.

The ARC experience also highlights the potential value of integrating rehabilitation with transitional care principles. Standalone hospital-to-home interventions and home-based management models have shown promise for improving patient-centred outcomes and reducing avoidable healthcare use in COPD^29 30^; however rehabilitation is not always embedded within these models in a structured way. ARC suggests these elements should not be conceptualised separately. The observed gains may have reflected the interaction between early inpatient mobilisation, structured assessment, coordinated discharge processes and ongoing follow-up, rather than any single component. From a service design perspective, this supports a shift toward pathway-based care where continuity and timing are treated as core therapeutic elements.

### Limitations

Several limitations need to be considered. First, the analytical cohort represented a subset of participants who engaged with the pathway and provided complete follow-up data (47 of 81 enrolled; ~58%). This is comparable to completion rates reported in similar post-AECOPD feasibility studies (38–74%).^45 46^ While baseline characteristics were broadly similar between groups, the analytical cohort likely reflects patients more able or willing to sustain engagement. Results should therefore be interpreted as outcomes among engaged participants within a real-world service model, rather than as effects generalisable to all AECOPD admissions.

Second, the uncontrolled, single-arm before-and-after design limits causal inference. The improvements observed over 8 weeks may reflect natural recovery after an exacerbation, regression to the mean, concurrent care and the structuring effect of ARC. The findings should therefore be interpreted as signals of improvement rather than estimates of treatment effect. A controlled or quasi-experimental evaluation will be needed to determine the added benefit of ARC compared with usual care. Third, ARC evolved across the three PDSA cycles, meaning that participants did not all receive an identical version of the pathway. This is expected in quality improvement work, but it makes interpretation more complex. Postdischarge support became more structured in later cycles through the shared tracking spreadsheet, home exercise booklet and RCCP safety huddles. Pooling data across cycles may therefore underestimate the effect of the more mature version of the pathway.

Fourth, this study focused mainly on functional and patient-reported outcomes. It was not designed or powered to robustly assess length of stay, readmissions, cost-effectiveness or broader healthcare utilisation. These outcomes are important, particularly if ARC is to be scaled or used to support workforce investment.

Fifth, long-term sustainability was not formally evaluated. Although later PDSA cycles identified workforce requirements and embedded several ARC processes within routine inpatient and community workflows, we did not prospectively assess intervention fidelity over time, apply a formal sustainability framework or measure longer-term adoption, penetration, maintenance or spread following the implementation period. Statements regarding sustainability therefore reflect implementation experience and observed resource dependence during the study rather than evidence that the programme was sustained over the longer term. Future evaluations should incorporate longitudinal fidelity and sustainability measures alongside clinical and healthcare-utilisation outcomes.

Finally, this was a single-centre study in a service with established respiratory physiotherapy expertise, access to a community respiratory follow-up programme and a culture of physiotherapy-led improvement. Implementation in other settings may require adaptation, particularly where inpatient gym space, senior respiratory staffing or community respiratory services are limited.

### Lessons learnt

- Pathway reliability matters as much as exercise content. Referral timing, documentation quality and cross-setting communication were major determinants of programme performance and should be treated as core implementation targets from the outset.
- Feasibility did not guarantee continuity. Early inpatient delivery was achievable before postdischarge adherence was reliably visible or supported.
- Structured measurement supports iterative improvement. Embedding PROMs and process tracking into routine eMR workflows enabled real-time gap identification.
- Student-supported delivery is a pragmatic adjunct, not a substitute for funded staffing. Feasibility depended on deliberate preparation, stable senior oversight and explicit attention to variability in student capability across placement cohorts.
- Inpatient and community rehabilitation must be designed and resourced as a single integrated pathway. Sustainable delivery of both phases is likely to require realistic, dedicated workforce capacity and should not rely on goodwill, excess capacity or short-term project funding.

## Conclusion

A physiotherapy-led integrated inpatient-to-community rehabilitation pathway for patients hospitalised with AECOPD can be implemented and iteratively refined through quality improvement methods within routine acute care. Among participants with complete follow-up, ARC was associated with clinically meaningful improvements in functional performance and health-related quality of life, with no exercise-related adverse events identified. The pathway offers a potentially useful model for improving rehabilitative continuity, but its effects on hospital avoidance, healthcare costs and long-term sustainability remain unestablished.

Further quasi-experimental or controlled evaluation should determine the pathway’s impact on length of stay, readmissions, broader hospital utilisation and cost-effectiveness, while prospective implementation evaluation should assess fidelity, adoption, maintenance, spread and the workforce resources required for sustained delivery.

## Supplementary material

10.1136/bmjoq-2026-004550 online supplemental file 1

10.1136/bmjoq-2026-004550 online supplemental file 2

10.1136/bmjoq-2026-004550 online supplemental table 1

## Data Availability

Data are available upon reasonable request. All data relevant to the study are included in the article or uploaded as supplementary information.
