# Supplementary material for: Implementing an integrated inpatient–community rehabilitation pathway for adults hospitalised with acute exacerbations of COPD: a quality improvement study
Source: BMJ Open Qual. 2026 Nov 10;15(4):e004550. doi: 10.1136/bmjoq-2026-004550 (PMC13756468; doi:10.1136/bmjoq-2026-004550)
Supplement: Supplementary file 1 [file bmjoq-15-4-s001.pdf]

Accelerated Respiratory Care (ARC)

PROGRAM PROTOCOL

Prince of Wales Hospital
Physiotherapy Department

1. Assessment

Assessing Exercise Capacity

1-Minute Sit-to-Stand Test (STST)

1-min Sit-to-Stand Test (1min-STST)

The one-minute sit-to-stand test is a valuable and valid alternative to 6MWT to estimate functional exercise performance in COPD patients.[2-4]

- A standard chair (height 46 cm) with no arm supports will be used.
- Patients will be instructed to stand up from and sit down on the chair with no support from the hands (arms crossed over the chest), repeating the procedure as many times as possible for the duration of 1-min at a patient defined pace.[5,6]
- After 45 secs patients are told "**you have 15 secs left until the test is over**".
- The test will first be demonstrated by a staff member and then performed by the patient.
- The number of stands during 1-min will be recorded manually

Interpretation:

With 6MWD as the anchor, the minimal important difference was found to be 2.5, showing 3 repetitions to be the improvement consistent with the physical benefits of a pulmonary rehabilitation program.[3] This result was recently validated by a multicenter study of Crook et al.,[6] who also considered 3 repetitions as the minimal important difference.

Assessing Lower Limb Function

Short Physical Performance Battery (SPPB)

3 SPPB calculation components:

1) Ability to stand for 10 sec with feet in 3 different positions (together side-by-side, semi-tandem, and tandem)
2) Two timed trials of a 3-m or 4-m walk (fastest recorded)
3) Time to rise from a chair five times

Scoring:

- Minimum score: 0
- Maximum score: 12
- Gait and chair subtests scored from 0-4, cut-off scores provided for scoring
- Higher scores indicating better lower extremity function

3 Balance Subsets:

- Side-by-side stand scored from 0-1
- Semi-tandem stance scored from 0-1
- Tandem stance scored from 0-2

Administration instructions: http://geriatrictoolkit.missouri.edu/SPPB-Score-Tool.pdf

Interpretation

COPD: (Bernabeu-Mora et al.[7]) n = 137; mean age = 66.9 (8.3) years)

Score of <10 indicates one or more mobility limitations.

Assessing Quality-of-Life & Patient Satisfaction

- **Barthel Index Dyspnoea (BI-D)**
- **modified Medical Research Council (mMRC) dyspnoea scale**
- **St. George's Respiratory Questionnaire (SGRQ)**
- **Depression Anxiety and Stress Scale (DASS-21)**
- **The Patient-Reported Outcomes Measurement Information System (PROMIS-29)**

Using the Health Outcomes and Patient Experience (HOPE) IT platform on admission, the following PROMs will be recorded at admission, pre-discharge, and 8 weeks post-discharge:

1. SGRQ
2. PROMIS-29

2. Inpatient ARC Exercise Protocol

1. **Warm up (10mins)**
2. **Lower Limb Endurance (20-30 min)**
3. **Upper Limb Endurance (10 min)**
4. **Strengthening (10 min) – intermittent days**
5. **Cool down (5 mins)**

1. Warm up, flexibility and stretching

Performed in standing or sitting

- Forward heel digs + punching out alternate arms
- Side toe taps + punching out alternate arms
- Knee lifts with opposite hand touching knee
- Hooks
- Upper cuts
- Karate chops
- Alternate back elbow strikes
- Seated marching + arm circles
- Kicks
- Toe taps + goal post
- Alternate punches forward + bilateral knee extension
- Alternate punches upwards + knee lifts

Breathing Exercises

- Pursed lip breathing
- Breathing control
- Rectangle breathing

Quadriceps stretch

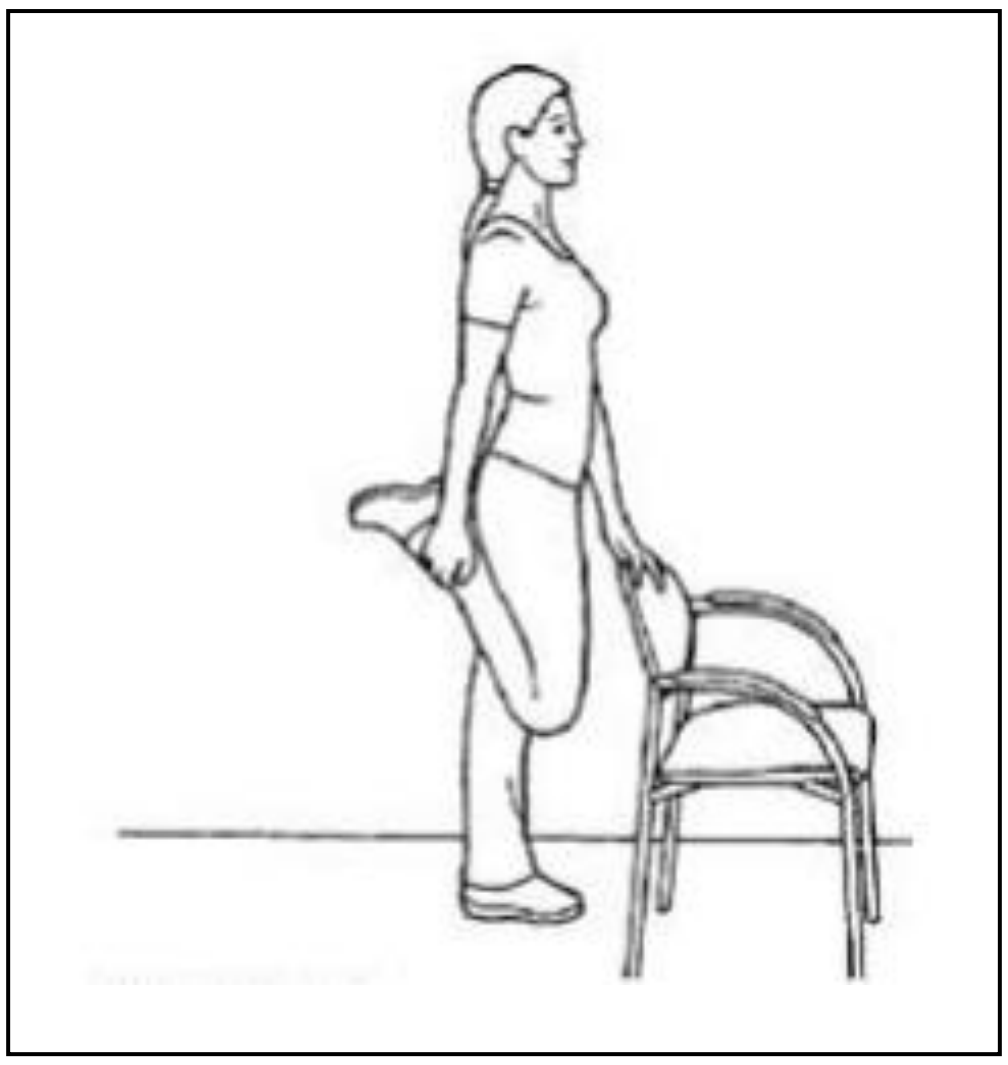

- Holding a chair / wall for support.
- Bend your right leg up behind you and hold onto either your ankle or back of your trousers.
- Try to take your foot towards your bottom and keep your knees together.
- You should feel a stretch at the front of the thigh.
- Return to normal position and relax.
- Repeat with the other leg.

Calf stretch

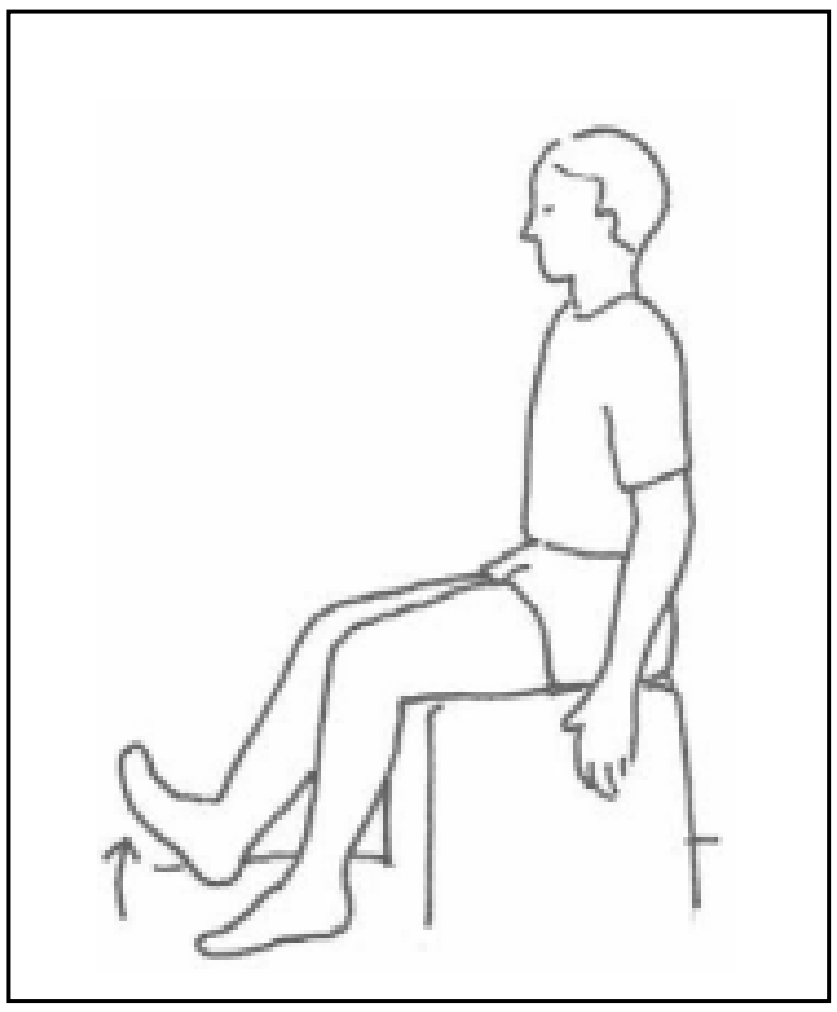

- Sit forward in a chair with your back straight.
- Stretch one leg up straight in front of you (keeping foot on floor).
- Keep your other leg bent.
- Stretch the toes of your straightened leg towards you.
- Return leg to floor and repeat with other leg.

Hamstring stretch

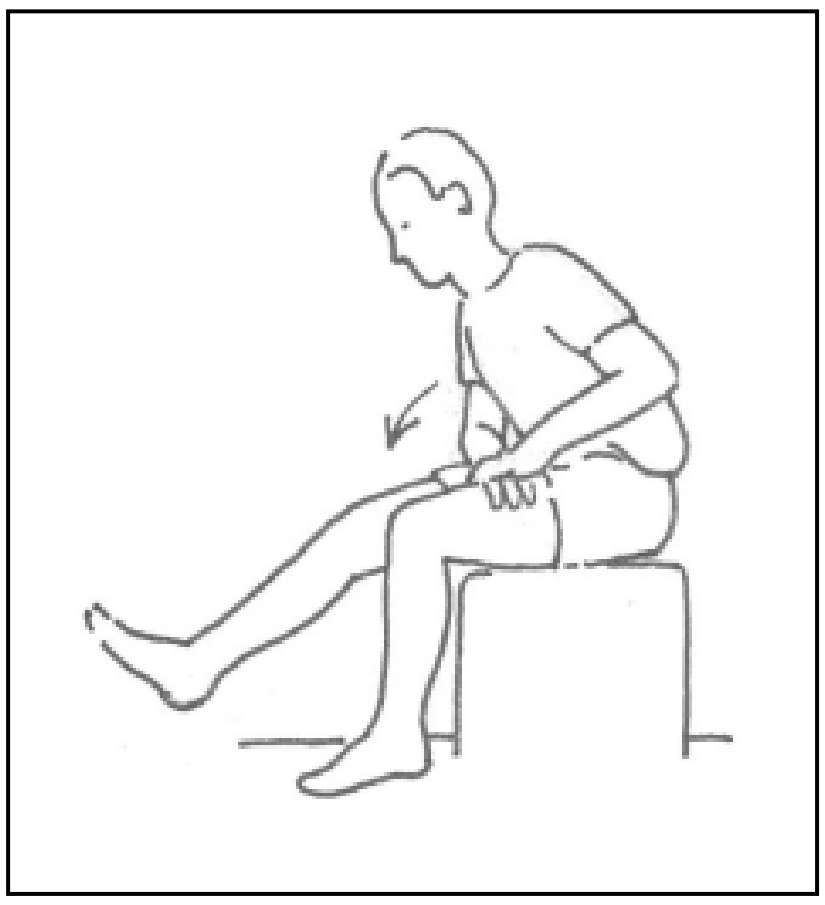

- Sit forward in a chair with your back straight.
- Stretch one leg up straight in front of you with the foot on the floor and relaxed.
- Put both your hands on your other leg, which should be still bent.
- Lean forwards from the hip, supporting your weight on your hands.
- You should feel a stretch at the back of the thigh of the straightened leg.
- Return to normal position and relax.
- Repeat with other leg

2. Lower Limb Endurance

MODE

Lower limb aerobic exercises (using large muscle mass):

- Walking training (over-ground or treadmill).
- Stationary cycling training

For patients with severe dyspnoea, fixing the shoulder girdle by using a wheeled walker (rollator) for walking training or leaning on to the handles when using a stationary cycle for cycle training allows:

- The accessory respiratory muscles to work more efficiently.
- This lean forward position may help to dome the diaphragm, improving its length/tension relationship

Note: These two strategies may result in a slight reduction in ventilatory constraints to exercise, allowing a greater work level to be achieved by the lower limb muscles e.g quadriceps, gastrocnemius and gluteal muscles.

For patients who experience oxygen desaturation during exercise, the level of oxygen desaturation is often less during cycling, compared to walking. However, it should be remembered that to achieve the greatest changes in a functional activity such as walking, it may be better to train in walking.

For people with **Bronchiectasis**, lower limb endurance exercise may facilitate movement of secretions. Encouraging airway clearance via huffing, or the patient's/individual's usual airway clearance routine following this exercise may be necessary. Other individuals with bronchiectasis may benefit from completing their airway clearance techniques prior to endurance exercise and should be advised accordingly.

While the optimal endurance training strategy for patients with **Interstitial Lung Disease** (ILD) still needs to be explored there is no evidence to suggest that the exercise prescription should vary from that provided to patients with COPD.

INTENSITY

Cycle training intensity:

Research shows that levels of dyspnoea in patients with COPD can equate to levels of oxygen consumption.[8] Therefore, the patient's level of dyspnoea may be used to monitor exercise intensity.

Most clinicians encourage their patients to exercise at a dyspnoea score of about 3-4 ("moderate to somewhat severe") on the modified Borg 0-10 scale as this equates to exercising at a cycle training intensity of approximately 75% VO2 peak. Therefore, patients could be encouraged to exercise at this level of dyspnoea.

The use of the dyspnoea score can help patients equate the level of exercise that they perform during the supervised exercise program to the level of exercise that they have been asked to perform during their home exercise program. If they maintain a similar level of dyspnoea, they should be exercising at a similar intensity.

Walking training intensity: It is typically recommended that for walking training a starting intensity should be 80% of the average six-minute walk test (6MWT) speed.

1. Exercise intensity for walking laps based on 6MWT

**To calculate an appropriate intensity for walking laps:<br>80% x ([6MWD ÷ 6] x prescribed duration)**
Six-minute walk distance (6MWD) ÷ 6 = Distance in one minute
For distance in 30 minutes = one minute distance x 30
For distance in 20 minutes = one minute distance x 20
For distance in 10 minutes = one minute distance x 10 etc...<br>The patient would not be expected to keep up the same walking pace throughout the walking<br>training session that they achieved in the 6MWT. Therefore, prescribe 80% of the calculated<br>distance by multiplying the prescribed duration distance by 0.8.

2. Exercise intensity for walking on a treadmill based on 6MWT

To calculate an appropriate intensity for walking on a treadmill:

Treadmill speed = 80% x (6MWT average speed)

Treadmill speed = 80% x 6MWT average speed

Treadmill speed= 0.8 x [(6MWD x10) ÷ 1000] = speed in km/hr

Note: On a treadmill the patient should be supervised and the speed adjusted by setting a slower speed initially until the patient has mastered the walking technique. Once the patient is confident and safe walking on the treadmill, increase the speed accordingly to the calculated walking speed.

DURATION

The recommended duration for a lower limb endurance exercise session is 30 minutes (e.g. 30 minutes of walking and/or cycling).

If a patient is very deconditioned the duration of the initial exercise sessions can be shortened (e.g. to 10 minutes with short rests if required). The duration should be built up towards 30 minutes in duration during the first two weeks of the program.

As a stationary cycle is available, the program can be split into 15 minutes of cycling and 15 minutes of walking.

TYPE

Exercise may be continuous or completed in an interval format.

- **Continuous training** is exercise at a prescribed intensity for the duration of the exercise period.

- **Interval training** is brief periods of high intensity exercise alternated with short periods of recovery (either rest or low intensity exercise). The total duration of exercise at the desired intensity still needs to be completed, therefore the duration of the exercise session will be longer (accounting for rests) than if the exercise was performed continuously.

- Interval training may be more suitable for patients who cannot sustain the prescribed intensity for the required duration of continuous exercise (i.e. due to severe dyspnoea, marked oxygen desaturation during exercise, signs of significant fatigue or presence of symptoms from co-morbid conditions, eg claudication pain). Interval training attenuates the fall in SpO2 typically seen with continuous exercise. People with ILD and COPD often prefer interval exercise training.[9-11]

Intermittent training may be needed in patients with severe disease or those who are very deconditioned. Intermittent training is short duration continuous exercise (e.g. 5 minutes) followed by a rest and repeated to build the prescribed duration.

3. Upper Limb Endurance Exercises

MODE

A Cochrane review of upper limb training in COPD has demonstrated that unsupported arm exercises had a large effect on unsupported arm endurance capacity.10 Consequently, a pulmonary rehabilitation program should include unsupported arm exercises with or without added weights (depending on patient's degree of dyspnoea and/or physical comorbidities).

Arm exercises should not cause significant discomfort to the shoulders or neck. Check with the patient about any shoulder or neck problems before commencing arm training and modify the range of movement if necessary. Cease arm exercises if shoulder or neck discomfort or pain persists.

(low weight and high repetition)

The exercises below were adopted from Ellis & Ries[12]:

Exercise 1

To be performed whilst seated in a chair with back support.

Sit with your feet flat on the floor, and the small of your back pressed back into the chair.

Elbows must not be locked.

Arms crossed in the lap, elbows straight and palms facing down.

Lift arms up, out and apart until they are fully extended above the head with palms facing backwards.

Return to starting position.

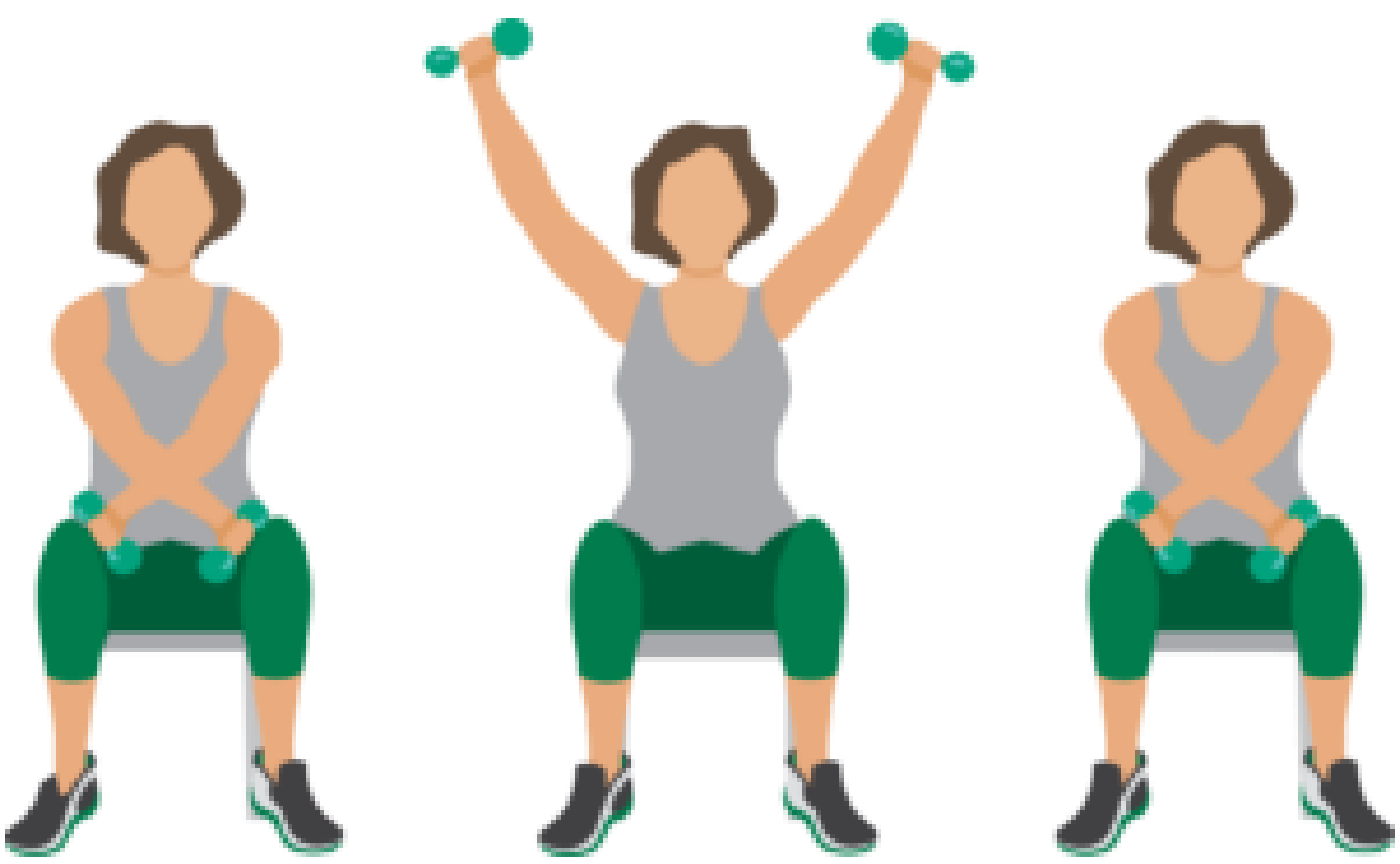

Exercise 2

To be performed whilst seated in a chair with back support.

Sit with your feet flat on the floor, and the small of your back pressed back into the chair.

Arms down by side, elbows straight and palms facing backwards.

Lift arms up, out and apart until they are extended above the head with palms facing backwards.

Return to seating position.

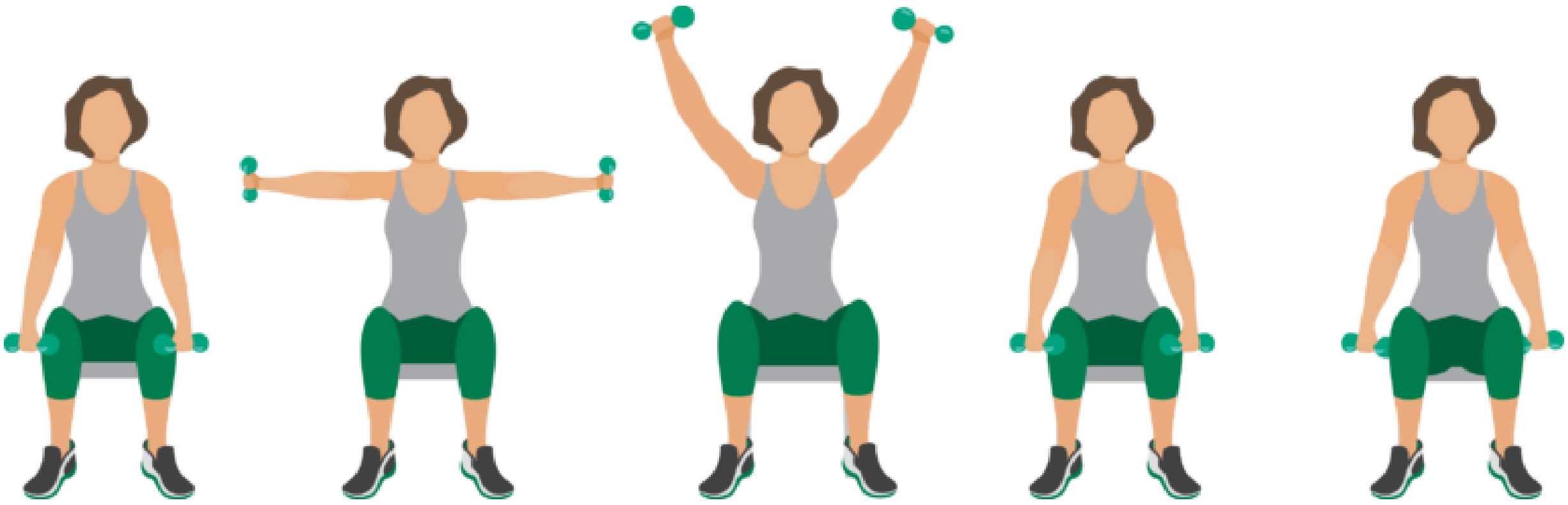

Exercise 3

Arms down by the sides, elbows straight and palms facing backwards.

Arms are lifted up and out to the side (horizontal) while rotating hands to finish with the palms facing forward.

Arms are moved forward to meet in the middle.

Return to starting position.

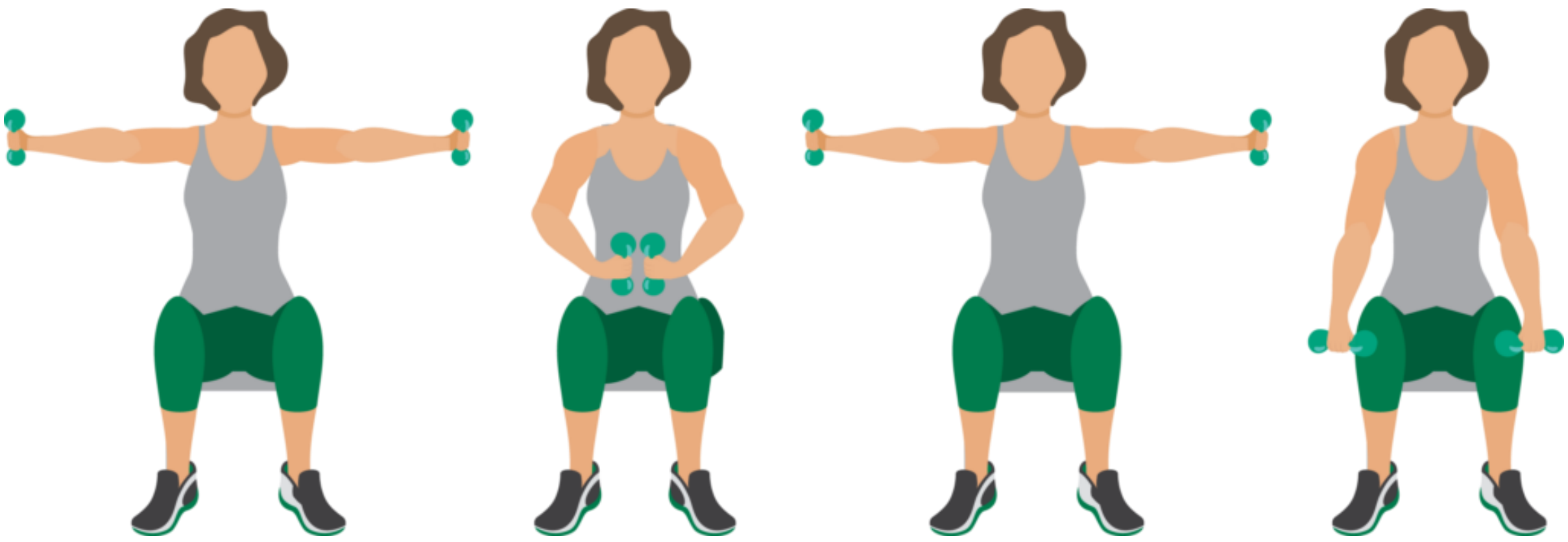

INTENSITY

Prescribing intensity based on weights and repetitions

To elicit an endurance training effect, use the upper limb endurance exercises, and prescribe low weights with high repetition.

Start with a weight that the patient can use to perform at least 12-15 repetitions of the chosen arm exercise (for some patients, the weight of their arms is sufficient as a 'starting weight').

After the patient can perform 15 repetitions of each exercise (one set) then increase to three sets of each exercise.

After the patient can perform three sets of each exercise, the weight held can be increased by 0.5 kg.

It may help to instruct the patient to move their arms up as they breathe in and down as they breathe out. However, if they find this difficult then just emphasise that the most important point is not to hold their breath.

It is generally recommended to perform the exercises in the in the sitting position with back supported. However, in everyday life upper limb activities are often carried out in standing. Some of the exercises can be performed in standing providing the patient can perform a correct technique without compensating with other body movements such as trunk flexion and extension.

DURATION

The duration of each upper limb endurance exercise session will depend on the number of sets the patient is able to achieve (15 repetitions of each exercise is one set).

If the patient is able to perform three sets of each of the three exercise, then the duration will be approximately ten minutes.

4. STRENGTH

An adequate strengthening program can be devised with or without portable (e.g. free weights or elastic bands) or fixed weight equipment and a person's own body weight may be sufficient stimulus. Exercises should be performed slowly and smoothly.

It is generally recommended to perform the arm exercises in the in the sitting position with back supported. However, in everyday life upper limb activities are often carried out in standing. Some of the exercises can be performed in standing providing the patient can perform a correct technique.

Examples of lower limb strengthening exercises without weight machines are listed below.

Lower Limb Strength Exercises

(high weight and low repetition)

**Exercise #1**<br>Knee extensions in sitting	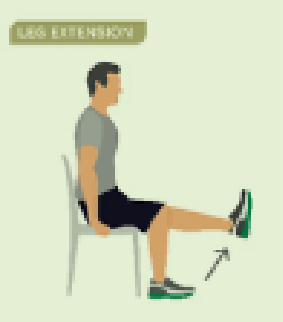	• Sit in a chair.<br>• Straighten your knee.<br>• Hold the knee straight for five seconds, and then relax.<br>• Repeat for other leg.<br>• Progression:<br>◦ Add ankle weights.
**Exercise #2**<br>Squats	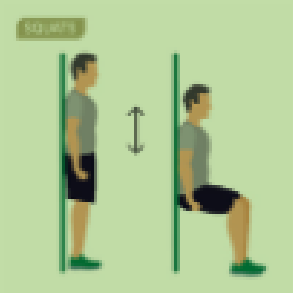	• Lean your back against a wall, you can use a fit ball between your back and wall to make it easier.<br>• Squat down until your thighs are parallel with floor (like you are sitting on a chair).<br>• Slide up the wall to a standing position.<br>• Start with only sliding down a short way.<br>• Progression:<br>◦ Increase depth of slide down.
**Exercise #3**<br>Climbing stairs or use a stepping box	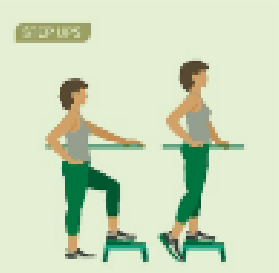	• Your instructor can help you with this exercise.<br>• Progression:<br>◦ Increase the number of steps.<br>◦ Increase the height of the step (or walk up two steps at a time).<br>• Carry a weight on your back using a backpack.

Upper Limb Strength Exercises

(high weight and low repetition)

Shoulder press	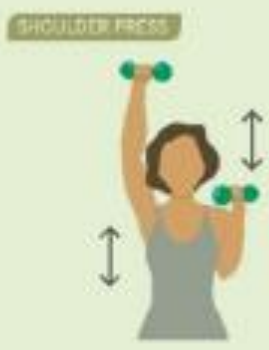	**The following exercises were adopted from:**<br>Lung Foundation Australia's *Better Living with Exercise- Your Personal Guide*<br>• Hold a weight in each hand at shoulder height.<br>• With one arm, lift the weight straight up and down.<br>• Breathe in while lifting the weight up, and breathe out while lowering the weight down.<br>• Repeat the exercise with your other arm.
Bicep curl	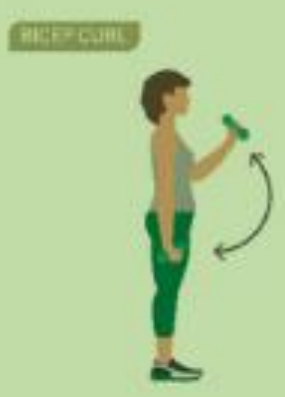	• Start with your arms by your sides.<br>• Bend your arm at the elbow to lift your hand towards your shoulder, then lower.<br>• Repeat the exercise with your other arm.<br>• Add hand weights as necessary.<br>• This exercise can also be done in the sitting position.
Wall press	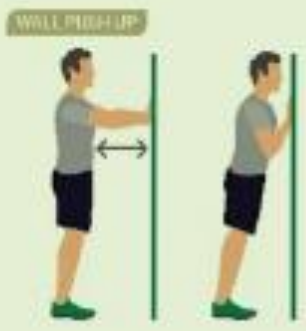	• Stand facing a wall with feet shoulder apart<br>• Leaning towards the wall place hands at shoulder height<br>• From the start position, bend your elbows moving your body forward<br>• Slowly push away from the wall and return to the starting position<br>• Ensure to keep body straight throughout

INTENSITY

Prescribing intensity based on weight repetitions

To avoid problems with testing patients for their 1 RM, the intensity of exercise may be prescribed in either of the following ways:

- Use a sub-maximal test to estimate the patient's 1 RM. The patient lifts a weight that they can only lift 2 to 3 times; this weight is taken as 80% of the patient's 1 RM. From this, the 1 RM can be calculated and the intensity prescribed as for the 1 RM method.
- Complete a 10RM test by following the same steps to performing a 1 RM test but working with 10 repetitions at a time and using this weight as the starting weight.

STRENGTH - PROTOCOL

- Perform one set (8-12 repetitions) of a particular exercise, then rest for 2-3 minutes.
- Once the patient can perform 2-4 sets of a particular exercise, the weight can be increased by 5% or between 0.5 kg to 5 kg depending on which muscle group is being trained (smaller increments for smaller muscle groups and larger increments for larger muscle groups). For upper body strength training, ask the patient to move their arms up as they breathe in, and down as they breathe out.
- Some exercises can be performed in the sitting position, with the back supported where possible.
- These exercises should not cause significant discomfort to joints.

The duration of a strength training session will be the time it takes to complete the appropriate number of sets.

3. RCCP Home-based Pulmonary Rehabilitation

The home-based exercise program:

- Start within 1 week of discharge home and delivered by the RCCP Physiotherapist.
- Home-based PR will be delivered x 2 per week for 8 weeks.

After the home-based pulmonary rehabilitation program is completed, patients should be encouraged to follow a home exercise program exercise for a total of 4 or 5 days per week. The home exercise program may include attendance at a supervised maintenance exercise class if available.

The home-based exercise training program will consist of:

- Walking for 30 minutes daily - at the same pace as during the pulmonary rehabilitation program (3 to 4 on the 1-10 Borg Dyspnoea Scale). For some people it is necessary to perform two or three smaller walks per day e.g., 10 minutes walking x 3, or 15-minutes walking x 2 with the aim to increase to continuous duration of 30 mins.

- Strength exercise (as a circuit) should be encouraged at least three days per week - similar exercises that are performed during the pulmonary rehabilitation program.

- Exercises, sets, reps, will be recorded by the RCCP physiotherapist in a patient exercise log book.

References

1. ATS Committee on Proficiency Standards for Clinical Pulmonary Function Laboratories (2002). ATS statement: guidelines for the six-minute walk test. Am J Respir Crit Care Med,166(1):111-7.

2. Reychler G, Boucard E, Peran L, Pichon R, Le Ber-Moy C, Ouksel H, Liistro G, Chambellan A, Beaumont M (2018). One minute sit-to-stand test is an alternative to 6MWT to measure functional exercise performance in COPD patients. Clin Respir J,12(3):1247-1256.

3. Vaidya T, de Bisschop C, Beaumont M, Ouksel H, Jean V, Dessables F, Chambellan A (2016). Is the 1-minute sit-to-stand test a good tool for the evaluation of the impact of pulmonary rehabilitation? Determination of the minimal important difference in COPD. Int J Chron Obstruct Pulmon Dis,11:2609-2616.

4. Vaidya T, Chambellan A, de Bisschop C. (2017) Sit-to-stand tests for COPD: A literature review. Respir Med, 128:70-77

5. Chorin F, Rahmani A, Beaune B & Cornu C (2015). Determination of reliable force platform parameters and number of trial to evaluate sit-to-stand movement. Aging Clinical and Experimental Research, 27, 473-482

6. Crook S, Büsching G, Schultz K, Lehbert N, Jelusic D, Keusch S & Frei A. (2017). A multicentre validation of the 1-min sit-to-stand test in patients with COPD. European Respiratory Journal, 49(3): 1601871.

7. Bernabeu-Mora R, Medina-Mirapeix F, Llamazares-Herrán E, García-Guillamón G, Giménez-Giménez LM & Sánchez-Nieto JM (2015). The Short Physical Performance Battery is a discriminative tool for identifying patients with COPD at risk of disability. International Journal of Chronic Obstructive Pulmonary Disease, 10, 2619-2626.

8. Zainuldin R, Mackey MG & Alison JA (2016). Prescribing Cycle Exercise Intensity Using Moderate Symptom Levels in Chronic Obstructive Pulmonary Disease. Journal of Cardiopulmonary Rehabilitation and Prevention, 36(3), pp. 195-202.

9. Dowman LM, May AK, Cox NS, Morris NR, Nakazawa A, Parker L, Bondarenko J, Holland AE (2021). Attenuation of exertional desaturation and preference for interval exercise compared to continuous exercise in people with interstitial lung disease. Respirology, 26(11):1076-1079.

10. Louvaris Z, Chynkiamis N, Spetsioti S, Asimakos A, Zakynthinos S, Wagner PD, Vogiatzis I (2020). Greater exercise tolerance in COPD during acute interval,

compared to equivalent constant-load, cycle exercise: physiological mechanisms. J Physiol, 598(17):3613-3629.

11. Wickerson L, Brooks D, Granton J, Reid WD, Rozenberg D, Singer LG, Mathur S. (2021) Interval aerobic exercise in individuals with advanced interstitial lung disease: a feasibility study. Physiother Theory Pract, 37(9):1034-1042

12. Ellis BPT & Ries AL (1991). Upper Extremity Exercise Training in Pulmonary Rehabilitation. Journal of Cardiopulmonary Rehabilitation and Prevention, 11(4), pp. 227-231.
