# Supplementary material for: Implementing an integrated inpatient–community rehabilitation pathway for adults hospitalised with acute exacerbations of COPD: a quality improvement study
Source: BMJ Open Qual. 2026 Nov 10;15(4):e004550. doi: 10.1136/bmjoq-2026-004550 (PMC13756468; doi:10.1136/bmjoq-2026-004550)
Supplement: Supplementary file 2 [file bmjoq-15-4-s002.pdf]

Prince Of Wales Hospital

Respiratory Coordinated Care Program (RCCP)

Supporting patients and their carers who are living with chronic respiratory disease

in collaboration with

Accelerated Respiratory Care (ARC) Program

Home Exercise & Walking Diary

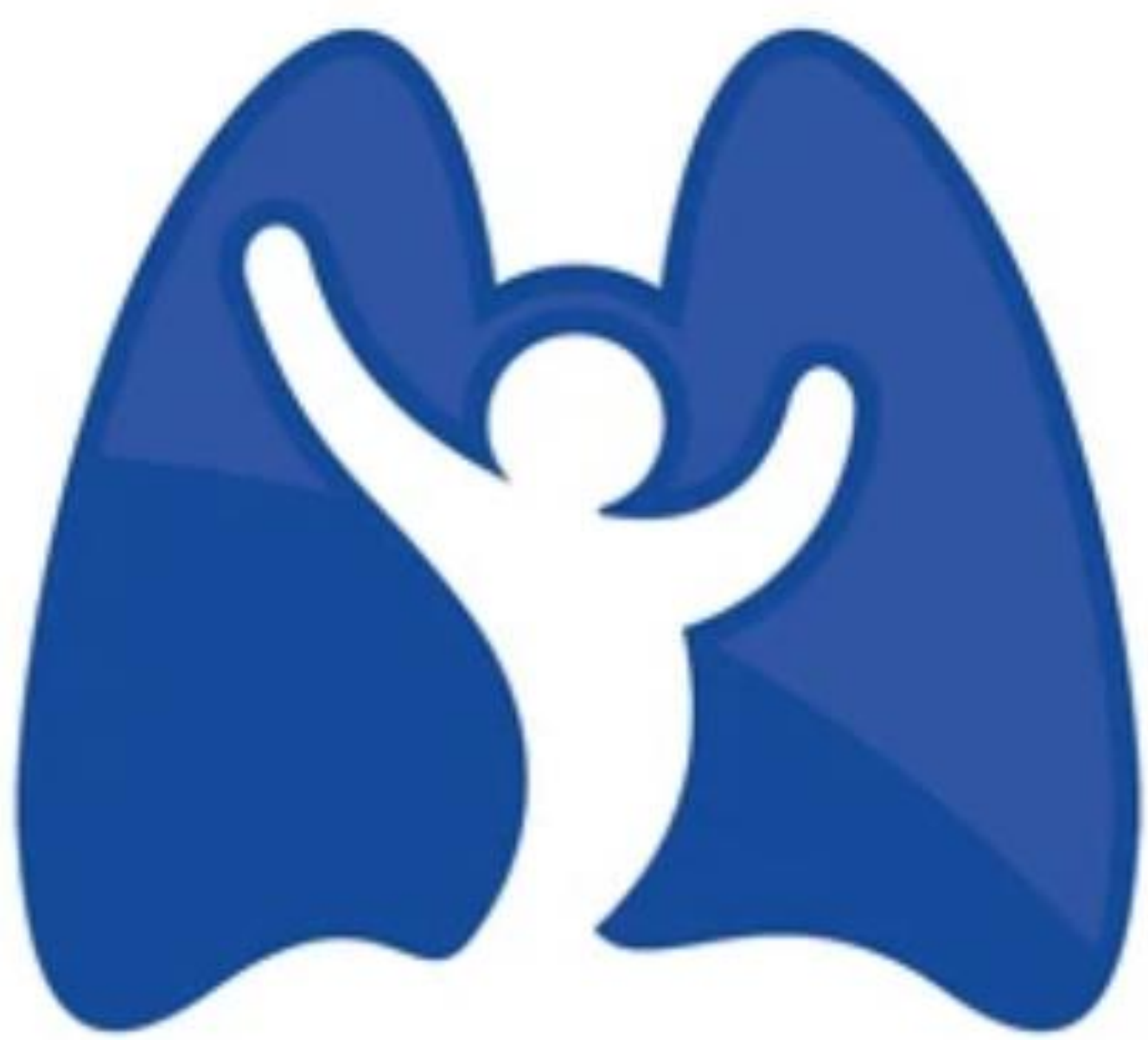

How Breathless Do you feel?

0	None
0,5	Very, very light
1	Very light
2	Light
3	Moderate
4	A little intense
5	Intense
6	
7	Very intense
8	
9	Very, very intense
10	Maximum

RCCP will help you:

- Manage symptoms of your chronic respiratory disease
- Understand your condition
- Improve your skills in managing your condition
- Understand how the healthcare system can help you
- Complete Accelerated Respiratory Care (ARC) program that you started in the hospital

ARC:

The goal is to provide patients with a structured and safe environment for their rehabilitation while you are in hospital and post discharge home for eight weeks.

In an emergency, always dial 000 and ask for an ambulance

Contact us:

Patients participating in the programme were provided with local RCCP contact details as part of routine clinical care. These details have been removed from the published supplementary material.

Hours of operation: Monday to Friday 8.00am - 4.30pm
Occasional weekend days

Check list:

Prior to discharge from hospital:

- Initial assessment form completed
- 8-week exercise program commenced

After discharge from hospital:

- Home visit review within 7 days post discharge by RCCP Physio
- Weekly contact from RCCP staff by phone *Home visit only if indicated
- 8 weeks post discharge review and completion of Physical assessment

Rehabilitation program included:

		Page
Warm up		7-8
Arm strengthening:	Exercise 1	9
	Exercise 2	10
	Exercise 3	11
Leg strengthening	Exercise 1	12
	Exercise 2	13
	Exercise 3	14
	Exercise 4	15
Cool down		16
Walking program		17-27
	Week 1 □	
	Week 2 □	
	Week 3 □	
	Week 4 □	
	Week 5 □	
	Week 6 □	
	Week 7 □	
	Week 8 □	

Managing our breathlessness with the 3Ps (pause, pursed lip, positioning)

Exercise becomes achievable by having control of your breathing with the “3 Ps”.

1. Pause

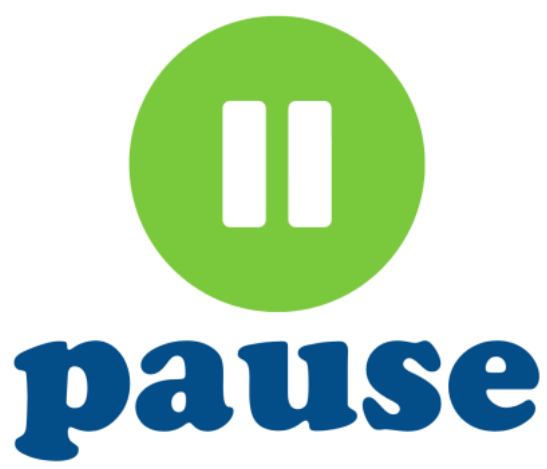

Remember:
When you feel short of breath, pause to remember that you can get out of this with the 3Ps

2. Pursed lip breathing

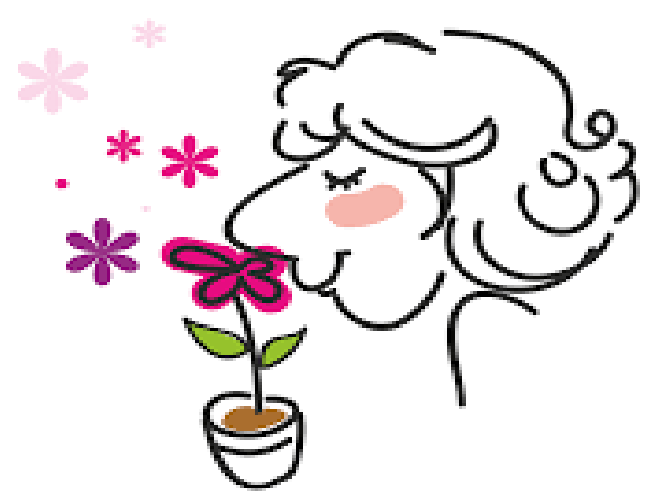

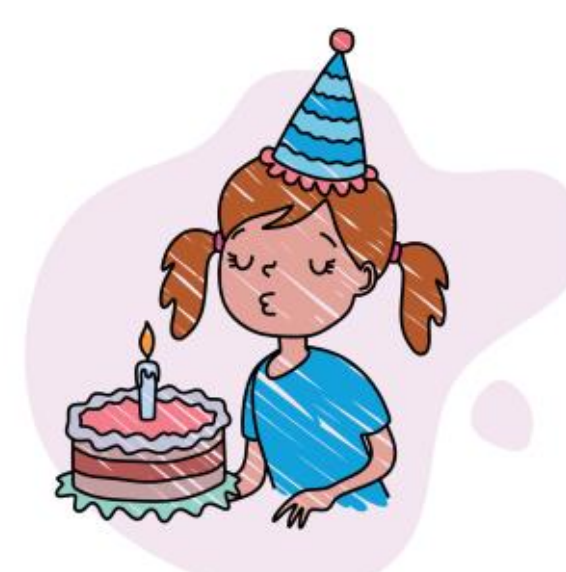

Remember:
Breathe in through the nose, “smell the roses”
Breathe out through pursed lips, “blowing out the candles”

3. Positioning

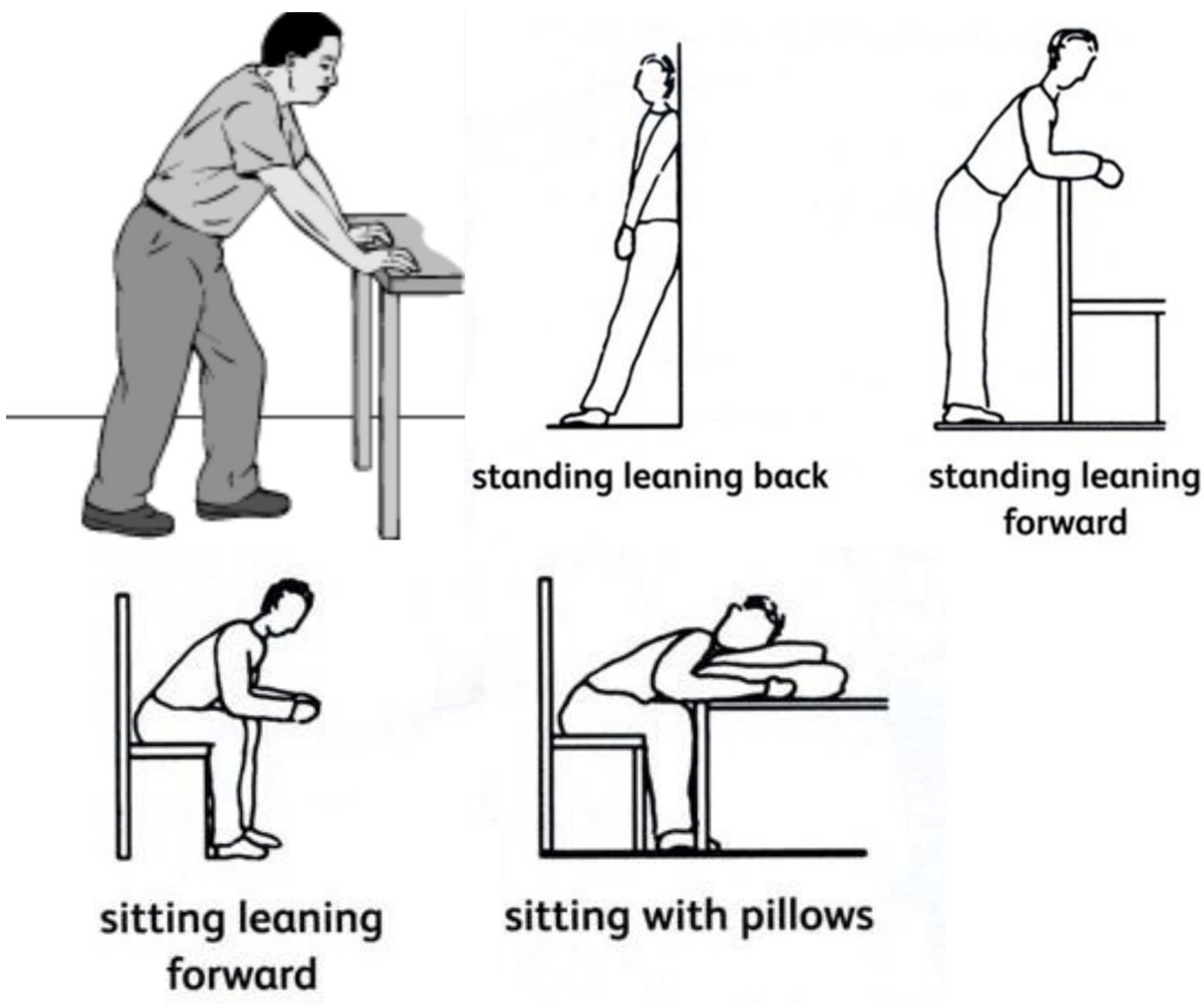

Remember:
Leaning forward on a chair, wall, table can make the breathing easier

Warm Up exercise

Shrugging the shoulders

1. Sit in a chair
2. Shrug the shoulders, hold for 3secs
3. Repeat 10 times ____ sets

Warm Up exercise

Marching on the spot

1. Sit in a chair
2. Lifting up one leg at a time, marching like a soldier in your chair
3. Repeat 10 times ______ sets

Arm Strengthening Exercise 1

Straight Arm Pull

1. Hold the band with your hand with palm facing down
2. The band should be nice and loose between your hands.
3. Pull the band and keep your arms straight
4. Hold the pull for 3 seconds
5. Repeat _____ times _____ sets

Arm Strengthening Exercise 2

Forearm pull

1. Hold the band with your hand with palm facing UP
2. Stretch the band, make sure your elbow is tucked in and against the side of your body
3. Repeat ______ times _____sets

Arm Strengthening Exercise 3

Diagonal elbow pull

1. Hold the band with your hand with palm facing down
2. Bring your right elbow to shoulder height
3. Straighten your right arm out as far as you can
4. DO this for _____ times ___ sets
5. Repeat step 1-4 on the left arm

Leg Strengthening Exercise 1

Leg Band Press

Looping the band on your foot

The movement

1. Loop the band under your foot

2. Pull the band against your foot with both of your hands
3. Keep your foot lifted off the floor
4. Bending your hips and knees
5. Then, straighten your leg against the band
6. Repeat _____ times _____ sets
7. Complete step 1-6 with your other leg

Leg Strengthening Exercise 2

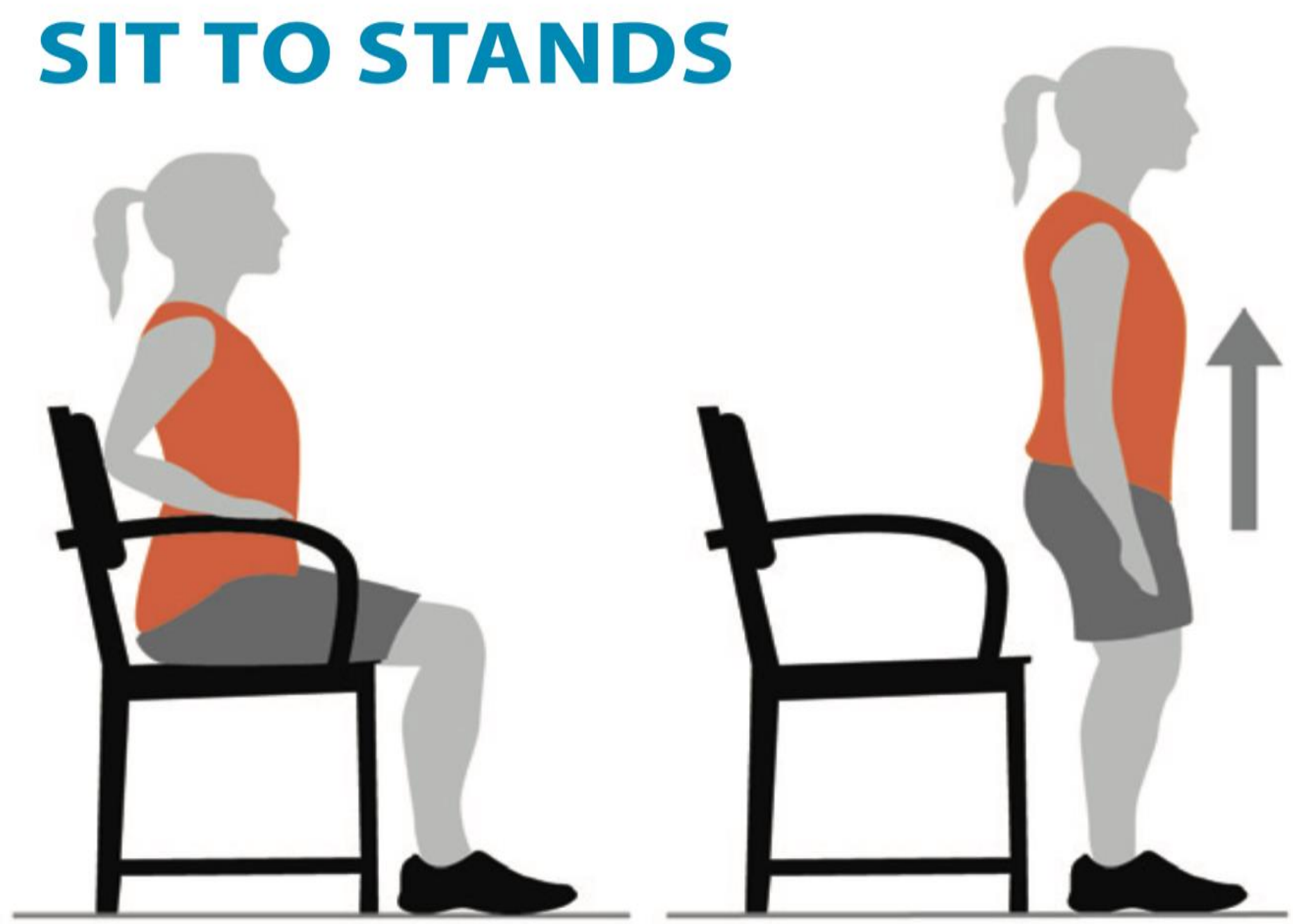

1. Ensure there is a chair behind you
2. Sitting down on the chair
3. Then, standing up straight. Try avoid using the handles if you can

4. Repeat ______ times ___ sets

Leg Strengthening Exercise 3

<u>Heel raises</u>

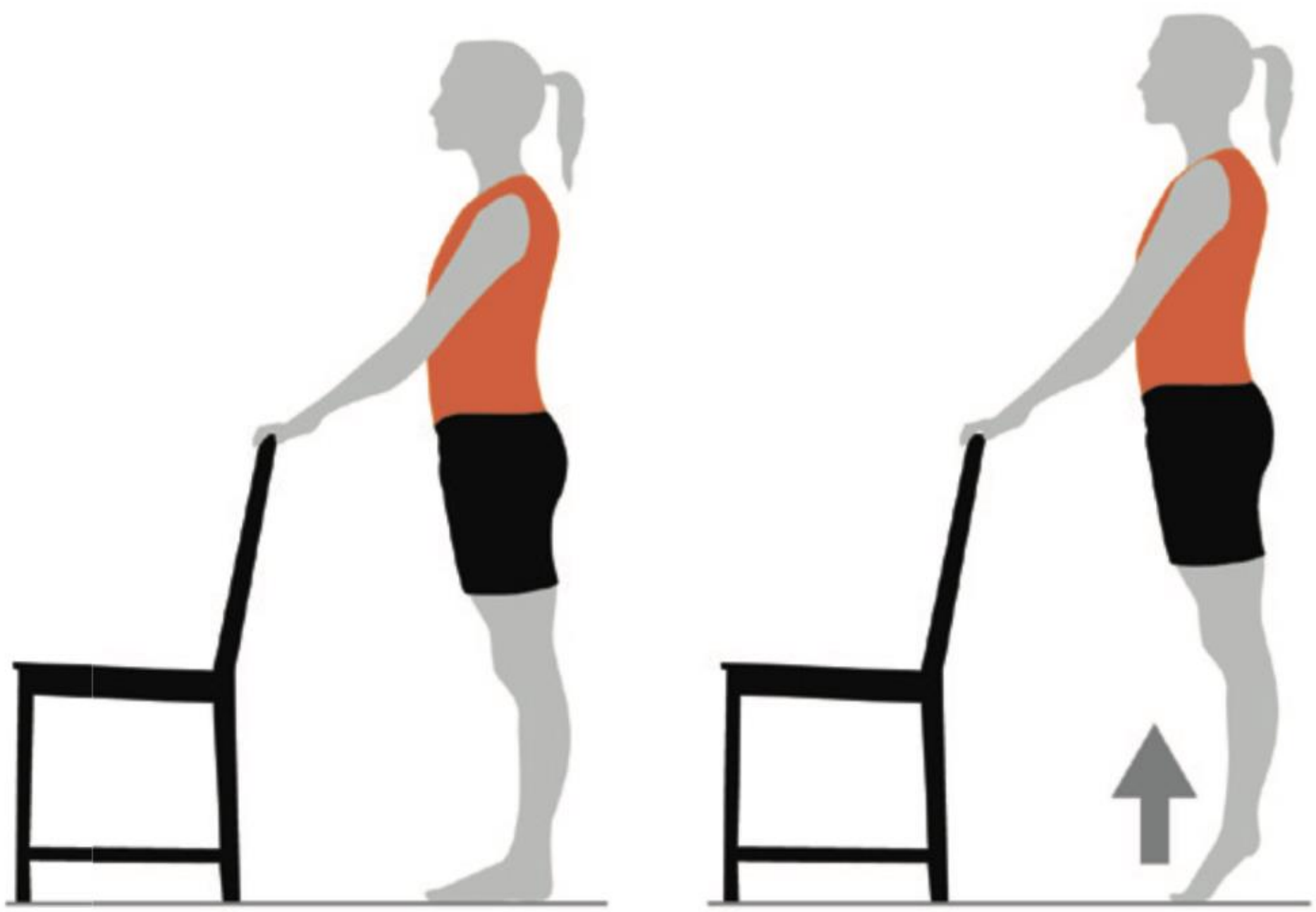

1. Make sure a chair is in front of you. If you can, only touching the chair with your fingertips.
2. Then slowly lifting both of your heels off the ground by going onto your toes.
3. Try to hold this position for ___ seconds.

4. Return to the ground by slowly lowering your heels.
5. Repeat _______ times _____ sets

Leg Strengthening Exercise 4

Chair Squats

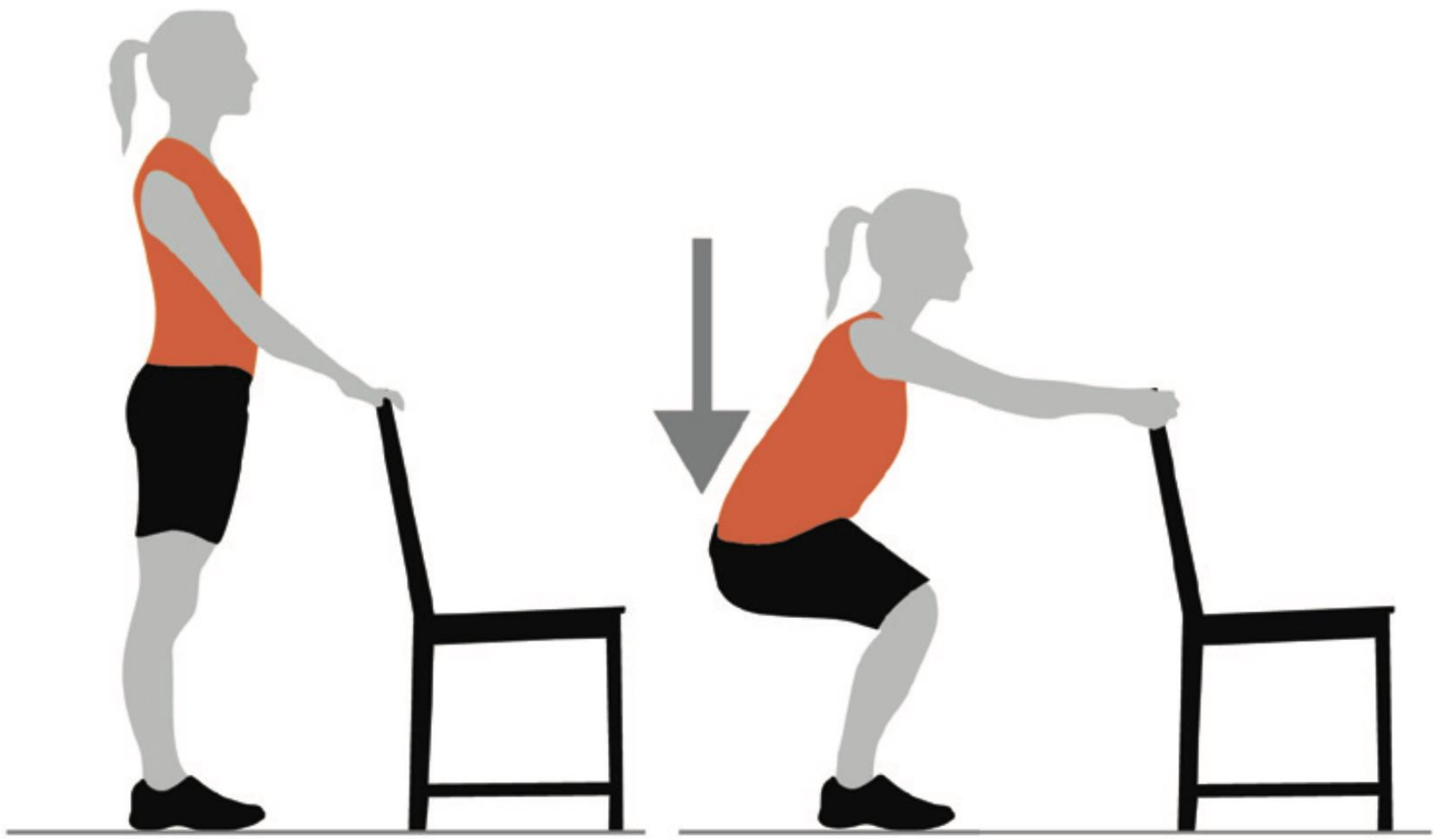

1. Make sure a chair is in front if you.
2. Then slowly bend your knees and lower your body towards the floor. Just like if you were sitting down on an imaginary chair.
3. Return to upright stand by straightening your knees

4. Repeat for ______ times ____ sets

Cool Down

Balancing

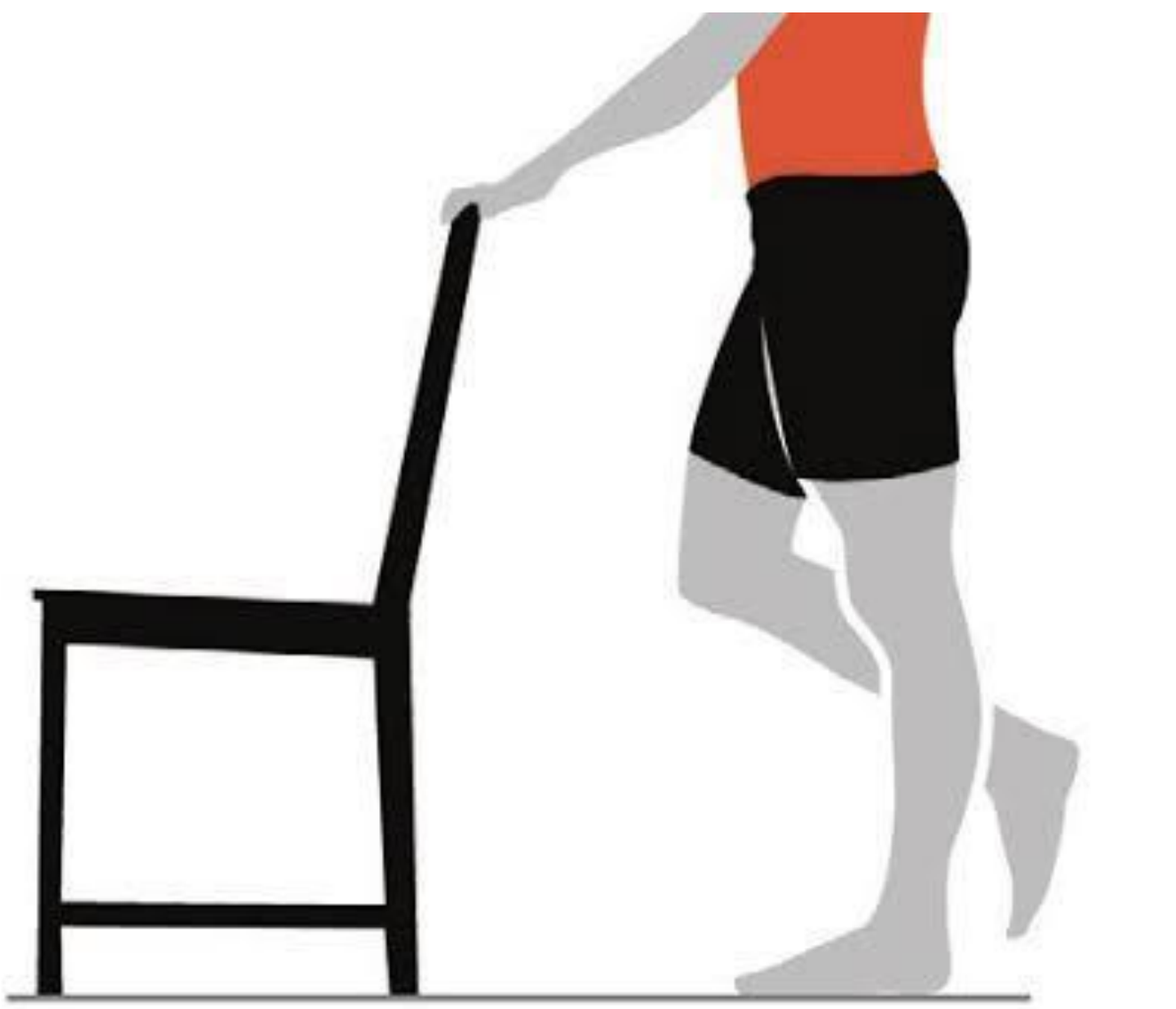

1. Make sure a chair is in front of you. If you can, only touch the chair with your fingertips.
2. Then slowly lifting one leg off the ground by bending your knees.
3. Try to maintain balance on one leg for 30 seconds.

4. Then repeat with the other leg. Aim for ____ repetitions.

Walking program

Managing our breathlessness with 3Ps

Walking becomes achievable by having control of your breathing with the “3 Ps”.

1. Pause

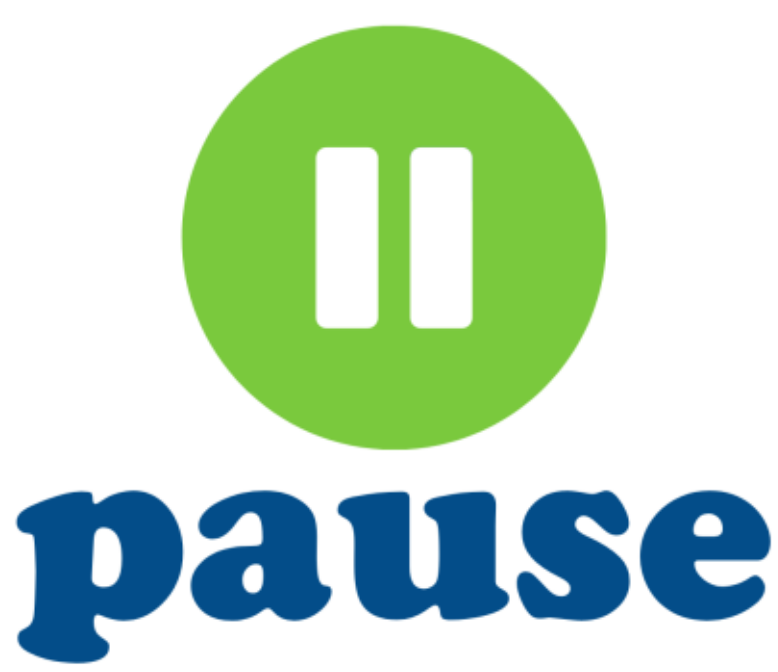

Remember:
When you feel short of breath, pause to remember that you can get out of this with the 3Ps

2. Pursed lip breathing

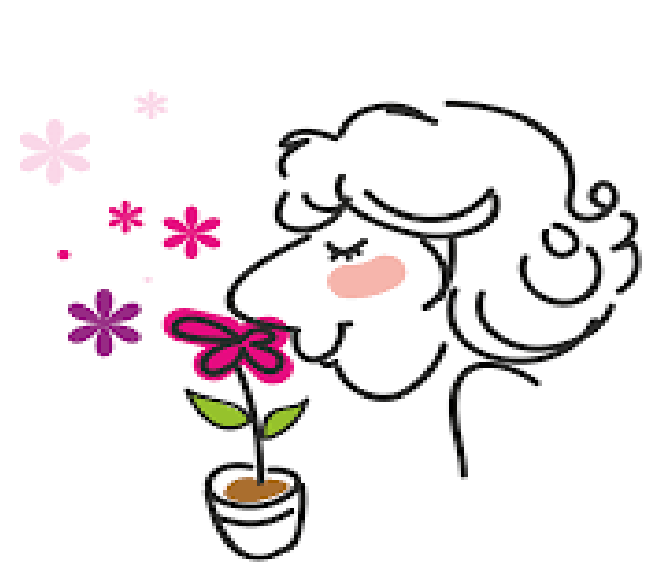

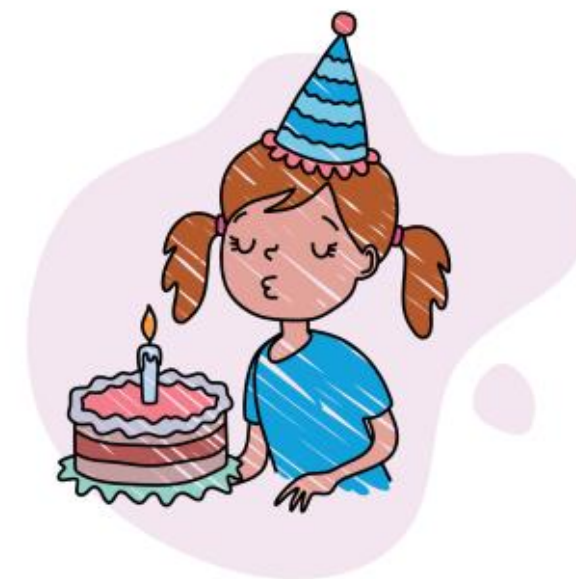

Remember:
Breathe in through the nose, “smell the roses”
Breathe out through pursed lips, “blowing out the candles”

3. Positioning

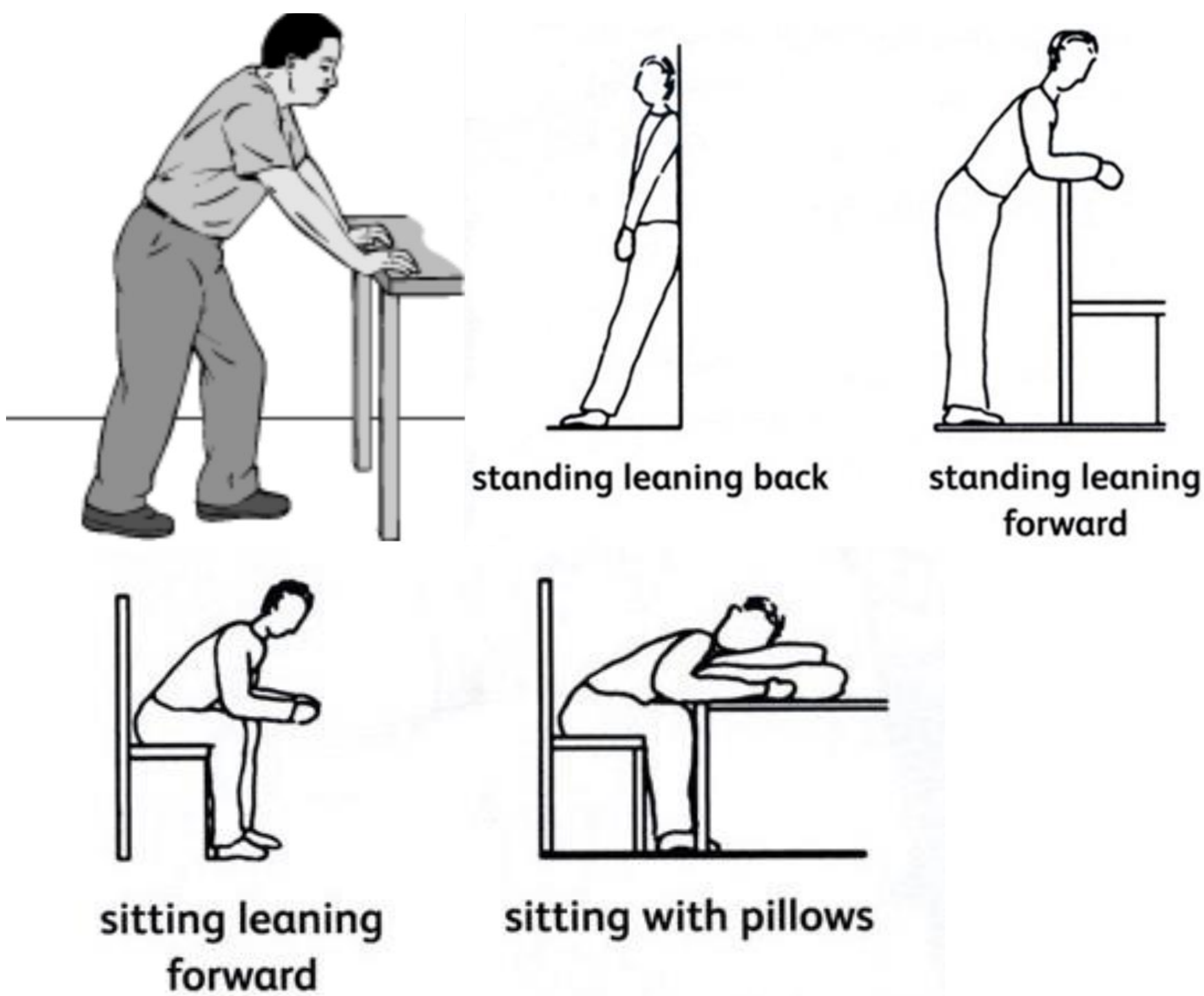

Remember:
Leaning forward on a chair, wall, table can make the breathing easier

Week 1 Walking and Exercise Diary

Date	Mon	Tue	Wed	Thu	Fri	Sat	Sun
Time (minutes)							
Number of rest							
Breathlessness (1-10)							
Strength exercise(sets)							

Walk _______ mins Exercise ______ sets

Week 2 Walking and Exercise Diary

Date	Mon	Tue	Wed	Thu	Fri	Sat	Sun
Time (minutes)							
Number of rest							
Breathlessness (1-10)							
Strength exercise(sets)							

Walk _______ mins Exercise ______ sets

Week 3 Walking and Exercise Diary

Date	Mon	Tue	Wed	Thu	Fri	Sat	Sun
Time (minutes)							
Number of rest							
Breathlessness (1-10)							
Strength exercise(sets)							

Walk _______ mins Exercise ______ sets

Week 4 Walking and Exercise Diary

Date	Mon	Tue	Wed	Thu	Fri	Sat	Sun
Time (minutes)							
Number of rest							
Breathlessness (1-10)							
Strength exercise(sets)							

Walk _______ mins Exercise ______ sets

Week 5 Walking and Exercise Diary

Date	Mon	Tue	Wed	Thu	Fri	Sat	Sun
Time (minutes)							
Number of rest							
Breathlessness (1-10)							
Strength exercise(sets)							

Walk _______ mins Exercise ______ sets

Week 6 Walking and Exercise Diary

Date	Mon	Tue	Wed	Thu	Fri	Sat	Sun
Time (minutes)							
Number of rest							
Breathlessness (1-10)							
Strength exercise(sets)							

Walk _______ mins Exercise ______ sets

Week 7 Walking and Exercise Diary

Date	Mon	Tue	Wed	Thu	Fri	Sat	Sun
Time (minutes)							
Number of rest							
Breathlessness (1-10)							
Strength exercise(sets)							

Walk _______ mins Exercise ______ sets

Week 8 Walking and Exercise Diary

Date	Mon	Tue	Wed	Thu	Fri	Sat	Sun
Time (minutes)							
Number of rest							
Breathlessness (1-10)							
Strength exercise(sets)							

Walk _______ mins Exercise ______ sets

Additional Information:
