# Supplementary material for: Implementing an integrated inpatient–community rehabilitation pathway for adults hospitalised with acute exacerbations of COPD: a quality improvement study
Source: BMJ Open Qual. 2026 Nov 10;15(4):e004550. doi: 10.1136/bmjoq-2026-004550 (PMC13756468; doi:10.1136/bmjoq-2026-004550)
Supplement: Supplementary file 3 [file bmjoq-15-4-s003.pdf]

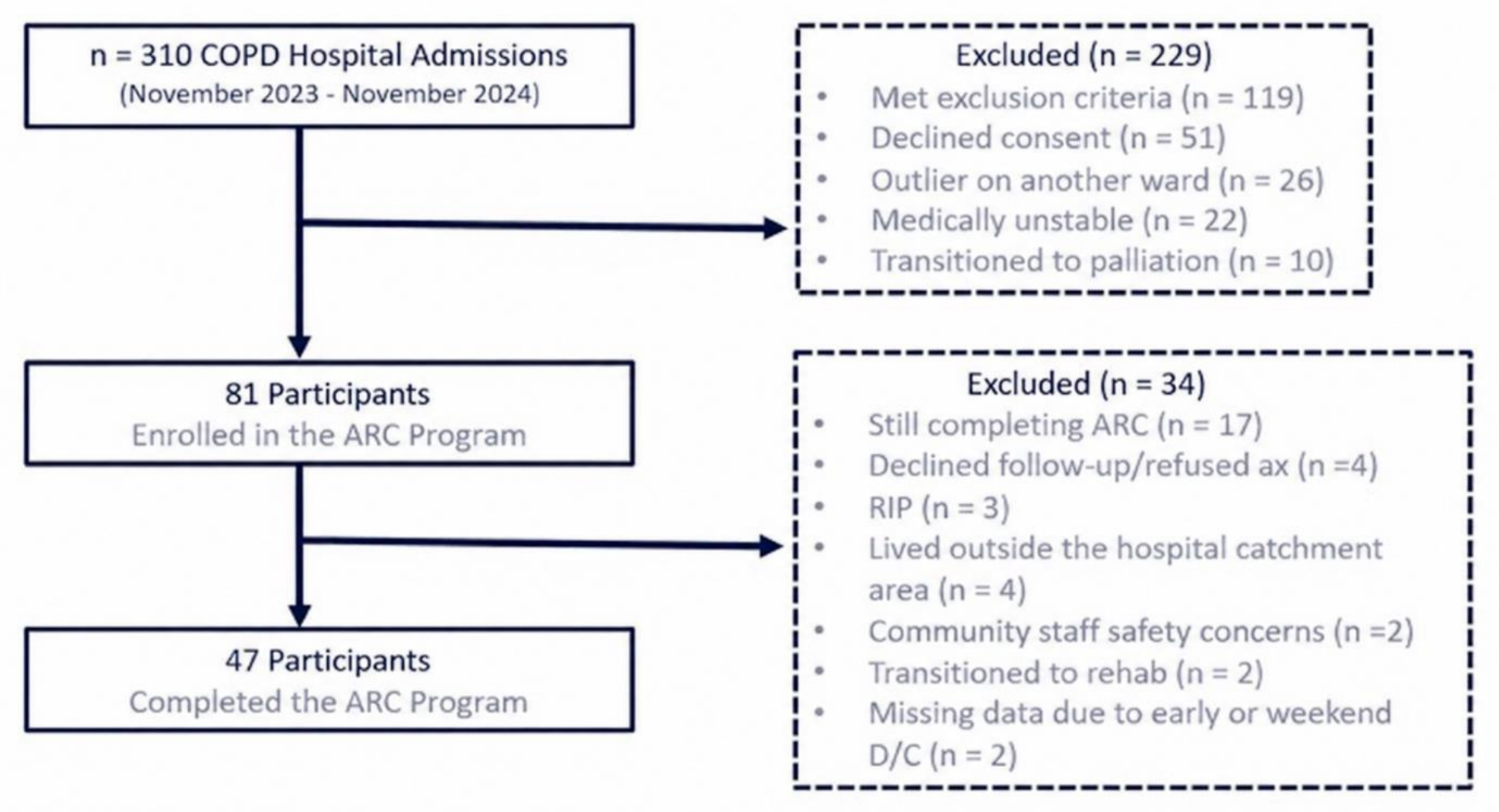

Supplementary Figure 1. Participant flow through the Accelerated Respiratory Care (ARC) programme. The final box represents participants with complete admission-to-8-week follow-up outcome data (n=47) included in the primary analysis.

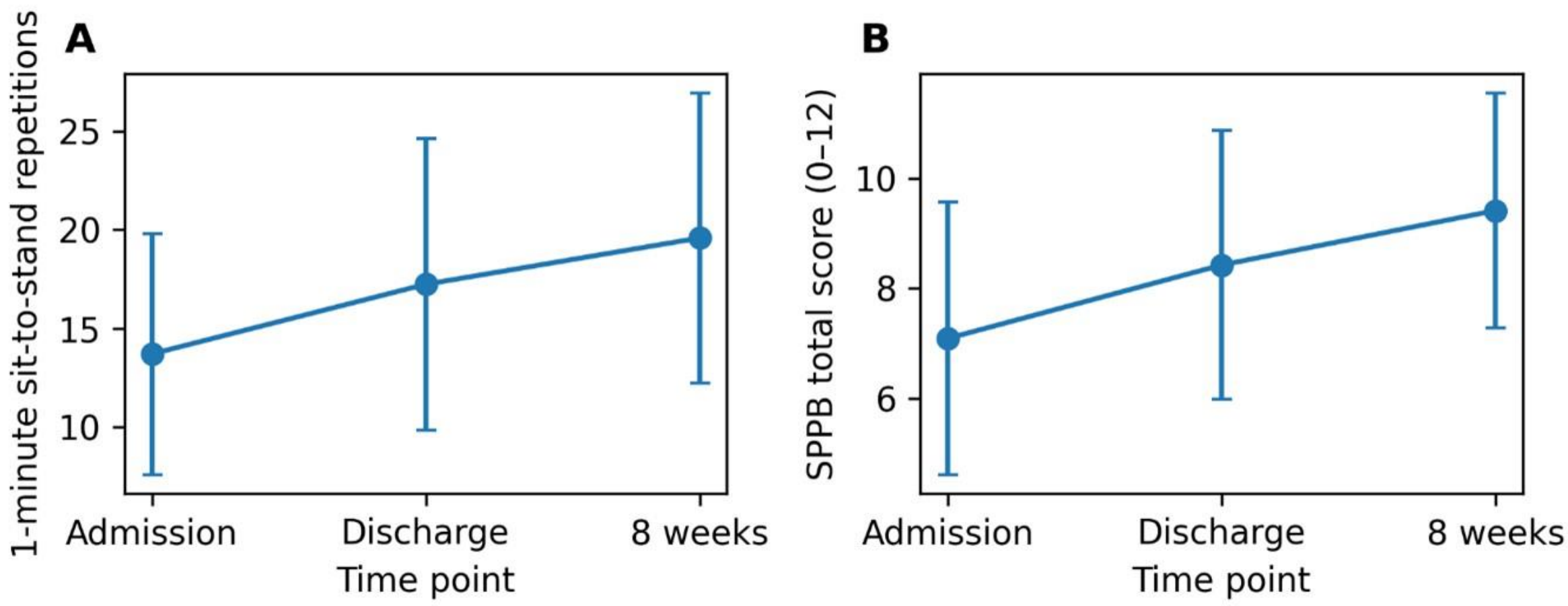

Supplementary Figure 2. Change in functional performance following participation in the Accelerated Respiratory Care (ARC) programme. (A) Mean one-minute sit-to-stand repetitions and (B) mean Short Physical Performance Battery (SPPB) total scores measured at admission, discharge, and 8 weeks post-discharge. Error bars represent standard deviations.

Supplementary Table 1. PDSA changes and associated evaluation data

PDSA cycle	Principal change ideas	Evaluation data	Implementation learning
**Cycle 1:** November – December 2023	Additional supervised gym-based rehabilitation delivered using existing staffing	Referral timing, attendance, inpatient completion and safety observations	Initial feasibility was established; referral and documentation processes required greater consistency
**Cycle 2:** January – August 2024	Dedicated gym space, standardised outcome measures, eMR and HOPE integration, formal RCCP referral processes and supervised student support	Inpatient participation, standardised outcomes, referral and transition data, and programme records	Inpatient delivery and measurement became more structured; community follow-up remained dependent on operational capacity
**Cycle 3:** September – November 2024	Shared ARC–RCCP tracker, home exercise booklet, enhanced discharge education and weekly review at the RCCP safety huddle	Community contacts, recorded exercise completion, follow-up completion and operational feedback	Visibility of post-discharge engagement improved; community resourcing remained a barrier

Abbreviations: ARC, Accelerated Respiratory Care; eMR, electronic medical record; HOPE, Health Outcomes and Patient Experience; PDSA, Plan–Do–Study–Act; RCCP, Respiratory Coordinated Care Programme.

Supplementary Table 2. Data collected during implementation and evaluation of the ARC pathway

Domain	Recorded variables	Timing/source	Use in evaluation
**Screening and enrolment**	Eligibility, enrolment, reasons for non-enrolment and first eligible admission	Screening records and eMR; during admission	Participant flow, denominator and enrolment
**Baseline characteristics**	Age, sex, BMI, COPD severity, comorbidities, previous hospitalisations and previous pulmonary rehabilitation	eMR; baseline	Cohort description and comparison of completers and non-completers
**Inpatient implementation**	ARC referral and commencement dates, time from admission to commencement, commencement within 72 hours, inpatient sessions attended and inpatient programme completion	ARC records and eMR; during admission	Referral timeliness, programme reach and inpatient participation
**Functional outcomes**	One-minute sit-to-stand test and Short Physical Performance Battery	Baseline, discharge and eight-week follow-up, as applicable	Within-participant changes in functional performance
**Patient-reported outcomes**	St George's Respiratory Questionnaire and PROMIS-29	HOPE and eMR; baseline, discharge and eight-week follow-up, as applicable	Within-participant changes in patient-reported outcomes
**Community implementation**	RCCP referral and transition, recorded community contacts and follow-up completion	RCCP records; following discharge	Continuity of care and recorded programme exposure
**Post-discharge monitoring**	Follow-up attendance, recorded exercise completion and follow-up contacts	Shared ARC–RCCP tracker; introduced during PDSA cycle 3	Identification of follow-up gaps and support for clinical review
**Healthcare utilisation**	Hospital length of stay and 28-day hospital readmission	eMR; index admission and 28 days following discharge	Evaluation of healthcare-utilisation outcomes
**Balancing measures**	Exercise-related adverse events and documented operational or workforce barriers	Clinical records and programme review; throughout implementation	Safety and implementation feasibility

ARC, Accelerated Respiratory Care; BMI, body mass index; COPD, chronic obstructive pulmonary disease; eMR, electronic medical record; HOPE, Health Outcomes and Patient Experience; PROMIS-29, Patient-Reported Outcomes Measurement Information System 29-item profile; RCCP, Respiratory Coordinated Care Programme.

The table reports measures documented during the evaluation and does not imply that session-level exercise intensity or duration, objectively verified home-exercise adherence, formal intervention fidelity, or long-term sustainability outcomes were collected.

Supplementary Table 3. Baseline characteristics, clinical course, and ARC programme participation: completers vs non-completers

Variable	Completers (n=47)	Non-completers (n=34)	p-value
**Demographics**			
Age, median (IQR), years	76 (69–83)	75 (68–81)	0.770
Female sex, n (%)	25 (53%)	17 (50%)	0.824
BMI, median (IQR), kg/m²	23.9 (20.9–28.0)	21.8 (18.3–26.5)	0.095
Smoking status, n (%)			0.192
Current	9 (19%)	11 (32%)	
Former	28 (60%)	20 (59%)	
Never	10 (21%)	3 (9%)	
**Lung function and COPD severity**			
Recent FEV_1 (% predicted), median (IQR)	40.0 (34.0–60.7)	39.0 (33.8–52.0)	0.363
GOLD COPD severity, n (%)			0.306
Stage 1 — Mild (FEV_1 ≥80%)	4 (9%)	0 (0%)	
Stage 2 — Moderate (FEV_1 50–79%)	10 (21%)	8 (24%)	
Stage 3 — Severe (FEV_1 30–49%)	23 (49%)	17 (50%)	
Stage 4 — Very severe (FEV_1 <30%)	2 (4%)	3 (9%)	
GOLD Stage 3–4, n (%)	25 (53%)	20 (59%)	0.656
**Comorbidities**			
Number of comorbidities, median (IQR)	2 (1–3)	1.5 (1–2)	0.101
Asthma, n (%)	14 (30%)	7 (21%)	0.444
Hypertension, n (%)	28 (60%)	20 (59%)	1.000
Coronary heart disease, n (%)	12 (26%)	2 (6%)	**0.035***
Dyslipidaemia, n (%)	15 (32%)	3 (9%)	**0.016***
Diabetes mellitus, n (%)	9 (19%)	7 (21%)	1.000
Obesity, n (%)	8 (17%)	5 (15%)	1.000
Stroke, n (%)	4 (9%)	5 (15%)	0.481
Heart failure, n (%)	4 (9%)	3 (9%)	1.000

Variable	Completers (n=47)	Non-completers (n=34)	p-value
**Medications and home oxygen support**			
Any inhaler use, n (%)	47 (100%)	34 (100%)	—
Inhaler therapy, n (%)			0.440†
LAMA only	0 (0%)	1 (3%)	
LABA + LAMA	8 (17%)	2 (6%)	
ICS + LABA	9 (19%)	4 (12%)	
ICS + LABA + LAMA	30 (64%)	27 (79%)	
Home oxygen use, n (%)	8 (17%)	4 (12%)	0.753
**Clinical course and rehabilitation history**			
ICU admission during encounter, n (%)	2 (4%)	0 (0%)	0.507
Length of hospital stay, median (IQR), days	7 (4–9)	5 (4–6)	0.147
28-day readmission, n (%)	9 (19%)	10 (29%)	0.301
Prior AECOPD hospitalisation in past year, n (%)	28 (60%)	13 (38%)	0.073
Previous pulmonary rehabilitation, n (%)	6 (13%)	3 (9%)	0.727
**Baseline functional performance**			
1-min STS at admission, mean (±SD), repetitions	13.7 (±6.1)	14.8 (±7.0)	0.478
SPPB total at admission, mean (±SD)	7.1 (±2.5)	7.7 (±3.0)	0.362
**ARC programme participation**			
Time to ARC commencement, mean (±SD), days	2.9 (±2.7)	2.9 (±2.4)	0.763
Time to ARC commencement, median (IQR), days	2 (1–4)	3 (1–4)	0.763
Commenced within 72 hours, n (%)	34 (72%)	22 (65%)	0.477
ARC inpatient sessions attended, median (IQR)	3 (2–3)	1 (1–2)	**<0.001***
ARC community RCCP contacts, median (IQR)	6 (3–10)	2 (0–6)	**<0.001***
ARC total sessions (inpatient + RCCP), median (IQR)	9 (5–12)	4 (2–6)	**<0.001***

p-values derived from Mann–Whitney U test for continuous variables and Fisher's exact test for binary categorical variables; chi-squared test for variables with >2 categories.
*p<0.05. †Chi-squared test across four inhaler categories.
AECOPD, acute exacerbation of chronic obstructive pulmonary disease; ARC, Accelerated Respiratory Care; BMI, body mass index; FEV_1, forced expiratory volume in 1 second; GOLD, Global Initiative for Chronic Obstructive Lung Disease; ICS, inhaled corticosteroid; ICU, intensive care unit; IQR, interquartile range; LABA, long-acting $beta_2$-agonist; LAMA, long-acting muscarinic antagonist; SPPB, Short Physical Performance Battery; STS, sit-to-stand.

Supplementary Table 4. Health-related quality of life outcomes at admission and 8 weeks post-discharge among ARC completers (n=47)

Measure	Domain	Admission Mean±SD	8 weeks Mean±SD	Mean Change	95% CI	p*
SGRQ	Symptoms	66.3±18.9	59.2±20.0	−7.1	−12.6 to −1.6	**0.013**
	Activity	76.2±19.5	71.5±23.3	−4.7	−10.6 to 1.2	0.118
	Impact	47.5±21.3	40.2±23.1	−7.3	−13.2 to −1.4	**0.016**
	Total‡	59.4±17.3	52.9±19.5	−6.5	−11.1 to −1.9	**0.006**
PROMIS-29	Physical Function†	34.0±7.0	36.2±7.4	+2.2	+0.7 to +3.7	**0.004**
	Anxiety‡	58.6±8.1	55.2±8.9	−3.4	−6.0 to −0.8	**0.011**
	Depression‡	55.1±7.6	52.0±8.3	−3.1	−5.7 to −0.5	**0.018**
	Fatigue‡	60.5±8.9	55.3±9.8	−5.3	−8.1 to −2.4	**0.003**
	Sleep Disturbance‡	59.3±8.7	56.4±9.5	−2.9	−5.7 to −0.1	**0.041**
	Social Roles†	41.2±8.9	44.8±9.2	+3.6	+1.1 to +6.1	**0.006**
	Pain Interference‡	61.5±8.4	58.1±9.0	−3.4	−6.1 to −0.7	**0.015**
	Pain Intensity§	5.2±2.1	4.4±2.0	−0.8	−1.5 to −0.1	**0.032**

SGRQ, St George's Respiratory Questionnaire; PROMIS-29, Patient-Reported Outcomes Measurement Information System.
*Paired t-tests comparing admission with 8-week follow-up.
†Higher scores indicate better function. ‡Lower scores indicate fewer symptoms.
§Pain Intensity scored 0–10; all other PROMIS-29 domains are T-scores (mean 50, SD 10).
